# Comparative Analysis of Osseointegration Using Titanium Implants Coated with Graphene and Niobium: An Animal Model Study

**DOI:** 10.3390/jfb17100525

**Published:** 2026-10-17

**Authors:** Marco Antonio Schueda, Charles Adriano Duvoisin, Diogo José Horst, Moisés Cohen

**Affiliations:** 1 Department of Orthopedics and Traumatology, Federal University of São Paulo (UNIFESP), São Paulo 04023-900, Brazil; marco.schueda@professor.unc.br (M.A.S.); m.cohen@uol.com.br (M.C.); 2 Department of Health Sciences, Italian University Institute of Rosario (IUNIR), Rosario S2002QUA, Argentina; charles.duvoisin@iunir.edu.arg; 3 Department of Mechanical Engineering and Materials, Federal University of Technology—Paraná (UTFPR), Curitiba 81280-340, Brazil

**Keywords:** osseointegration, titanium implants, diamond-like carbon, niobium-containing surfaces, coating delamination, rabbits

## Abstract

Background: Surface modification may influence tissue–implant interactions. This exploratory study compared the biomechanical, coating-integrity, and histological responses associated with uncoated titanium and carbon- or niobium-containing surface-processing conditions in a rabbit model. Methods: Twelve adult male New Zealand White rabbits were initially enrolled and allocated to 30- and 60-day healing periods. Two animals were lost before endpoint and were not replaced; ten rabbits completed the study and were analyzed, with five animals at each period. Each rabbit received four implant-site conditions: uncoated titanium, carbon-based diamond-like carbon (Graphene-DLC), niobium deposited by chemical vapor deposition (Nb-CVD), and niobium deposited by sol–gel processing (Nb-Gel). Implant stability was assessed using reverse-torque testing after formalin fixation. Histological evaluation included hematoxylin and eosin and Picrosirius Red staining. Results: At 60 days, the uncoated titanium and Graphene-DLC conditions showed the highest reported mean reverse-torque values, whereas some niobium-containing conditions showed lower reported coating-integrity scores. Ordinal histological outcomes were interpreted descriptively because the sample was small, implant sites were clustered within rabbits, and implant condition was fixed to anatomical site. Conclusions: Under this protocol, the observed differences describe combined implant-site conditions and do not provide unbiased causal evidence of coating-specific effects. The findings are exploratory and support further studies using randomized or counterbalanced allocation, clustered analysis, and independent biomechanical and histological specimens.

## 1. Introduction

Nanotechnology is a cornerstone of modern medicine, involving the manipulation and control of matter at atomic and molecular scales. Its application in healthcare—often referred to as nanomedicine—is derived from the fundamental principles of nanoscience.

Due to its excellent biocompatibility, titanium remains the gold standard for implants. Current research increasingly focuses on surface functionalization to optimize bone–tissue interaction and durability. However, the release of titanium oxide debris from orthopedic prostheses raises toxicity concerns, necessitating the development of preventive coating strategies to enhance long-term interfacial stability and biological safety [1,2,3,4,5].

The surface functionalization of biomaterials using graphene and niobium-based materials has emerged as a high-performance strategy for enhancing osseointegration. Graphene derivatives facilitate osteogenesis by increasing surface area and protein adsorption, thereby accelerating mesenchymal stem cell differentiation. Integration of niobium oxide further enhances the electrochemical stability and biocompatibility of the implant. Moreover, this composite coating exhibits potent antimicrobial activity, effectively preventing peri-implant infections through physical membrane disruption and reactive oxygen species (ROS) generation, ensuring a sterile and regenerative interface [6,7,8,9].

The synergistic potential of integrating graphene with metal oxides as a dual-action approach to combating peri-implantitis is investigated by several authors. This combination not only optimizes the biological response at the bone–implant interface by promoting osteogenic differentiation but also establishes a robust barrier against bacterial colonization. This “dual-action” mechanism relies on graphene’s ability to serve as a nanostructured scaffold that enhances the controlled release of metal ions and generates localized oxidative stress, effectively disrupting pathogen membranes without compromising the viability of mammalian cells. This strategy represents a significant advancement in surface engineering, offering a multifunctional solution to increase the longevity of dental and orthopedic implants in clinically challenging environments [10,11,12].

The primary objective of this study was to describe the biomechanical, coating-integrity, and histological responses associated with the four assigned implant-site conditions over 30 and 60 days. Because implant condition was fixed to anatomical site and the number of animals was limited, the study was considered exploratory and was not designed to provide an unbiased causal comparison of coating chemistry.

## 2. Materials and Methods

### 2.1. Materials and Implant Specifications

Grade IV titanium implants (7 × 2.5 mm, Cone Morse model—CM SCREW PLATINUM—Koppe Implants, Curitiba, Brazil) were utilized in this study. The surface-coating procedures and characterizations were conducted at the CVD Coating—São José dos Campos, SP Brazil and Department of Materials Engineering from the Federal University of Santa Catarina (UFSC)—Florianopolis, SC Brazil.

### 2.2. Coating Methodology: Diamond-like Carbon (DLC)

The deposition of DLC films was performed using a magnetron sputtering system equipped with a high-vacuum chamber and pumping assembly. A Dual Pulsed Sputtering (DPS) source was employed to generate negative bias pulses with a constant amplitude at 160 kHz, modulated by a 2.5 kHz frequency. The power delivered to the plasma was regulated by the pulse-per-unit-time ratio. The magnetron sputtering configuration was optimized to maximize the effective power transmitted to the graphite target, thereby enhancing sputtering efficiency.

The surface groups are identified according to their complete deposition conditions. Graphene-DLC refers to the carbon-based DLC condition produced using a graphite target in an Ar/CH_4_ atmosphere, with Raman and XPS findings used to support the presence of graphitic or graphene-like carbon-related features. Niobium-CVD refers to the niobium-containing CVD condition prepared with Nb_2_O_5_ and graphene oxide nanoparticles. It therefore represents a niobium oxide/carbon-containing processing condition rather than a pure metallic-niobium coating. Coating thicknesses were reported separately because thickness may influence mechanical integrity, delamination, and tissue response.

Deposition experiments were executed under two distinct power regimes: low effective power (70 W/0.15 A) and high effective power (140 W/0.2 A). A 5 cm diameter high-purity graphite disk served as the sputtering target. The deposition process was carried out in a controlled atmosphere consisting of an Argon (Ar) and Methane (CH_4_) gas mixture, regulated by mass flow controllers. The optimized deposition conditions were established at an Ar/CH_4_ ratio of 1:4, maintaining a total working pressure between 0.5 and 0.7 Pa. The substrate temperature range was 150–300 °C using a specialized ceramic heating plate, with temperatures monitored via a thermocouple coupled directly to the sample holder. Under these specified parameters, the average deposition rate was approximately 3 nm/min, resulting in a final DLC coating thickness of approximately 5 µm on the titanium substrates. The methodology for depositions was based on the study of Marciano et al. (2011) [13].

The analyzed surfaces were characterized using Raman spectroscopy and X-ray photoelectron spectroscopy (XPS). The spectra presented in this study were obtained from reference specimens produced under the same respective coating conditions as the experimental implants; they were not acquired from the implanted devices themselves. These analyses therefore provide chemical and vibrational information about the corresponding surface-processing conditions but do not document post-implantation changes in the individual implants.

**Figure 1:**
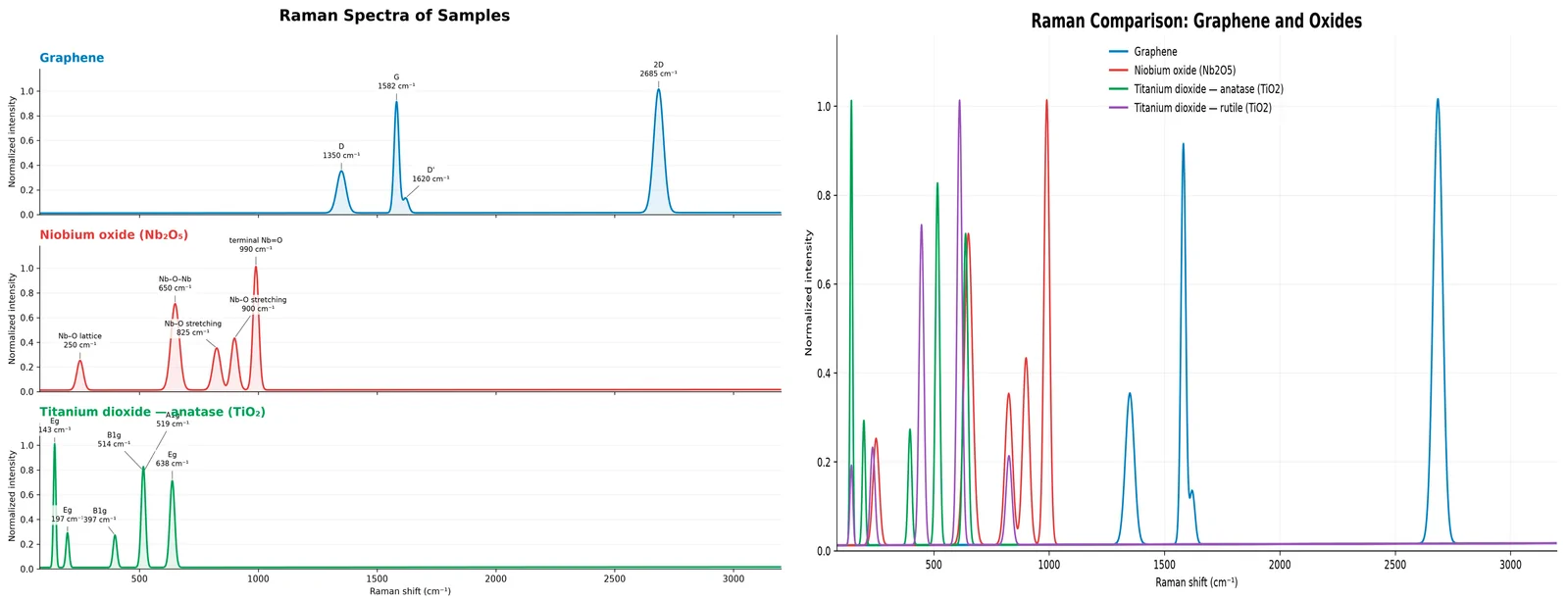
Raman spectra of samples analyzed.

Raman spectroscopy was performed using a Renishaw inVia Qontor confocal Raman microscope equipped with a thermoelectrically cooled CCD detector and a 532 nm laser excitation source. Spectra were collected at room temperature using a 50× microscope objective over the range of approximately 100–3200 cm^−1^. The laser power at the sample was maintained between 1 and 10 mW to minimize local heating and possible modification of the coatings. The spectrometer was calibrated using the Raman band of crystalline silicon at 520.7 cm^−1^. Spectra were acquired with an integration time of approximately 10–60 s and one to three accumulations per measurement. Data acquisition and processing were performed using WiRE 5.6 software. Raman spectroscopy was used to identify the characteristic vibrational features associated with carbon-based structures, niobium oxide, and titanium oxide phases (Figure 1).

**Figure 2:**
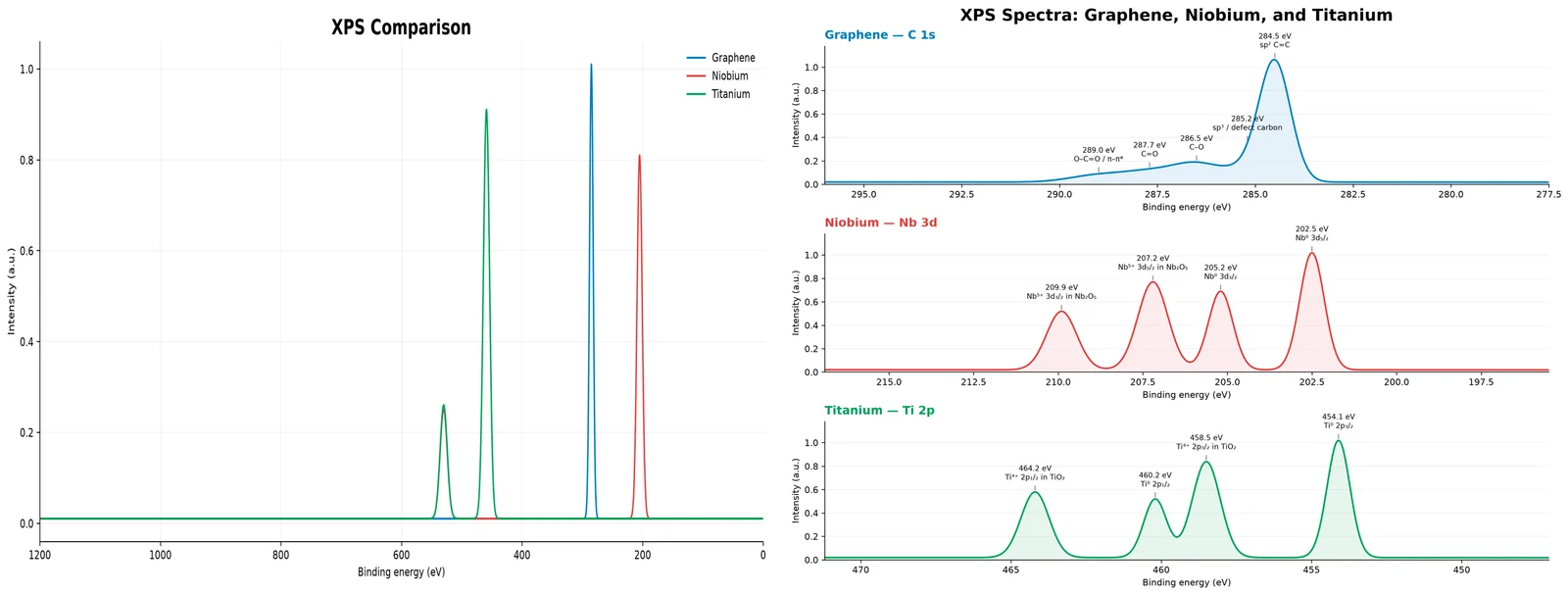
XPS spectra of samples analyzed.

X-ray photoelectron spectroscopy was performed using a Thermo Scientific K-Alpha+ spectrometer (Thermo Fisher Scientific, Waltham, MA, USA) equipped with a monochromatic Al Kα X-ray source operating at a photon energy of 1486.6 eV and a hemispherical electron analyzer. The measurements were performed under ultrahigh-vacuum conditions, with an operating pressure of approximately 10^−9^ mbar. Survey spectra were acquired over the binding-energy range from 0 to 1350 eV, followed by high-resolution scans of the C 1s, O 1s, Nb 3d, and Ti 2p regions. The analyzed area was selected using an X-ray spot size between approximately 30 and 400 µm, according to the sample geometry. The binding-energy scale was calibrated using the adventitious carbon C 1s peak at 284.8 eV. Charge compensation was applied when necessary. Spectra were processed using Thermo Advantage software (Thermo Fisher Scientific, Waltham, MA, USA; version not recorded in the original experimental records) with background subtraction and peak fitting to identify the chemical states of carbon, oxygen, niobium, and titanium (Figure 2).

The Raman and XPS results were used to support the chemical characterization of the corresponding surface-processing conditions and to assess carbonaceous and oxide-related species. Because the analyses were performed on reference specimens rather than on the implanted devices, they do not establish the post-implantation state of the individual surfaces. The characterization also did not include a complete evaluation of the surface morphology, coating thickness, roughness, adhesion strength, or particle release. These limitations are relevant when interpreting coating stability and delamination.

### 2.3. Chemical Vapor Deposition (CVD) Coating

Niobium pentoxide (Nb_2_O_5_) films were separately deposited using a Rapid Thermal Processing (RTP) furnace (Annealsys RTP system). A crucible containing 10 g of Nb_2_O_5_ and graphene oxide (GO) nanoparticles (5–100 nm) was placed within the quartz tube. The system was preheated to 750 °C, reaching a peak deposition temperature of 950 °C. Nitrogen (N_2_, 1000 mL/min), Hydrogen (H_2_, 10 mL/min), and Methane (CH_4_, 5 mL/min) were utilized as carrier and cleaning gases for the drag deposition process, following the methodology described by Horst et al. (2021) [14].

### 2.4. Sol–Gel Coating Procedure

**Figure 3:**
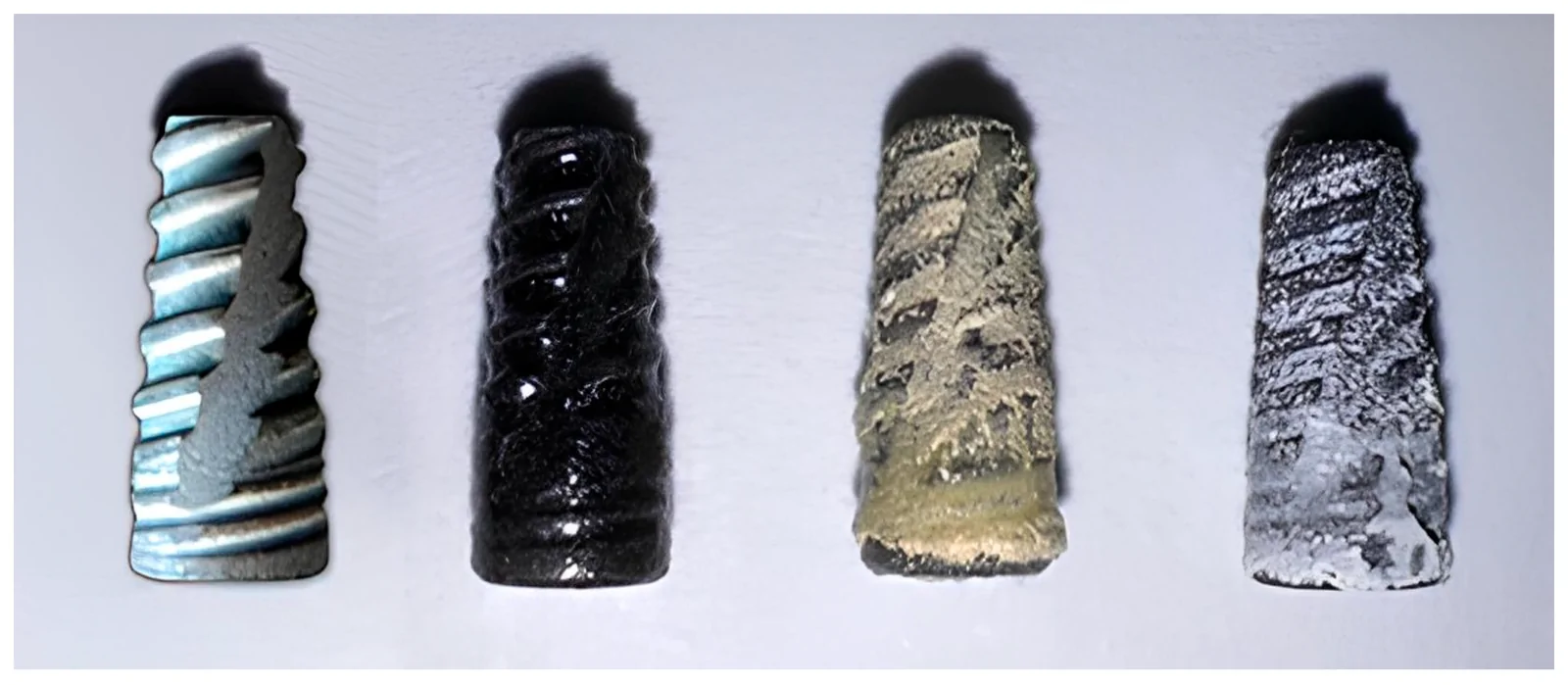
Macro-photograph of implants from left to right: without coating (pure Ti); Graphene-DLC; Niobium-CVD; niobium sol–gel.

The synthesis of the coatings was performed using the sol–gel technique, employing a colloidal suspension of niobium nanoparticles (5–100 nm) as the primary precursor. This suspension was incrementally introduced into 2 mL of acrylic acid and subsequently diluted in a mixture of 20 mL of distilled water and 10 mL of ethanol. To ensure uniform dispersion and prevent nanoparticle agglomeration, the solution underwent ultrasonic processing (sonication) for 90 min. The titanium implants were then submerged in Petri dishes containing the prepared sol. The assembly was placed in a rotary evaporator and maintained at 180 °C for 24 h to achieve complete solvent evaporation and facilitate the gel-to-solid transition. Following this, the coated samples were thoroughly rinsed and subjected to a drying stage at 70 °C for 2 h, following the protocol established by Safavi et al. (2022) [9]. The resulting niobium-based films exhibited a thickness range between 450 nm and 2 µm. Figure 3 shows the implants.

### 2.5. Surgical Procedure and Implant Methodology

Following an established animal model protocol, four Grade IV titanium implants (7 × 2.5 mm, Cone Morse model—CM SCREW PLATINUM—Kopp Implants, Curitiba, Brazil) were placed into the femoral condyles of each subject (rabbit). The experimental design included one uncoated control implant and three groups featuring the nano-coatings. The implants are shown in Figure 3.

Implant sterilization: Before implantation, the titanium implants and coated specimens were sterilized before surgery. Detailed cycle parameters and post-sterilization surface characterization data were not available for the present retrospective analysis; therefore, no inference is made regarding the effect of sterilization on coating morphology or adhesion.

### 2.6. Animal Model and Ethical Considerations

Adult male New Zealand White rabbits, aged 6–12 months and weighing 2.8–3.5 kg, were included in the study. The animals were housed individually in climate-controlled facilities with ad libitum access to water and a standardized diet. All surgical procedures were performed at the Nursery Vivarium of the University of Contestado, Mafra Campus. The experimental protocol was approved by the Institutional Animal Care and Use Committees (CEUA/UNC and CEUA/UNIFESP; protocol numbers 03/24 and 9704270225, respectively).

### 2.7. Experimental Groups and Clinical Attrition

Twelve adult male New Zealand White rabbits were initially enrolled and allocated to the 30-day and 60-day healing periods, with six animals assigned to each period. Two animals were lost before the scheduled endpoint and were not replaced: one because of a bone fracture and one because of death of unknown etiology during the early postoperative period. Ten rabbits completed the study and were included in the final analysis, with five animals at 30 days and five at 60 days. Each rabbit received all four implant-site conditions in the split-body design. Accordingly, the animal was the independent biological unit, the final animal-level sample size was *n* = 5 per healing period, and the four implant sites within each rabbit were treated as clustered observations rather than independent biological replicates. No a priori power calculation was performed.

### 2.8. Anesthesia and Preoperative Protocol

The animals were initially weighed to calculate precise dosages. Pre-anesthetic medication (PAM) was administered intramuscularly (IM) and consisted of a combination of Midazolam (4 mg/kg), Acepromazine 0.2% (1 mg/kg), and Tramadol (2 mg/kg). Once a stable pre-anesthetic plane was achieved (approximately 30 min post-administration), general anesthesia was induced via IM injection of Xylazine 2% (5 mg/kg) and Ketamine (35 mg/kg).

### 2.9. Surgical Technique and Implant Distribution

**Figure 4:**
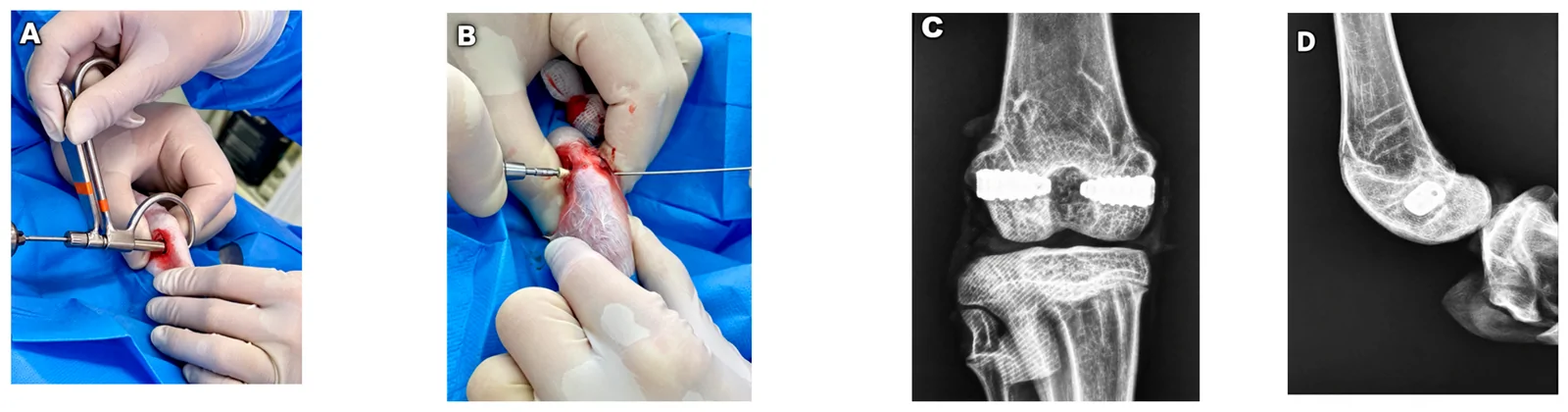
Surgical implantation procedure [14] and radiographic verification of implant positioning. (**A**) Transcondylar preparation of implant bed using surgical guide and 2.5 mm drill under continuous saline irrigation. (**B**) Placement and fixation of implant in prepared femoral condyle. (**C**,**D**) Postoperative anteroposterior and lateral radiographic views documenting implant positioning.

Animals were identified using ear tags. After trichotomy, asepsis, antisepsis, and sterile draping, a lateral knee incision was performed, and a surgical guide was used to standardize implant-bed preparation. Transcondylar drilling was performed with a 2.5 mm drill at low rotational speed under continuous sterile saline irrigation. The implant was inserted into the prepared bed according to a standardized protocol [15]. All procedures were performed by the same surgeon. Guide-wire placement and final implant position were documented radiographically (Figure 4).

The implants were assigned to the anatomical sites as follows:

- Right lateral condyle (RL): uncoated titanium control implant.
- Right medial condyle (RM): Graphene-DLC-coated implant.
- Left medial condyle (LM): Nb-CVD-coated implant.
- Left lateral condyle (LL): Nb-Gel-coated implant.

### 2.10. Postoperative Care and Clinical Monitoring

Daily postoperative care consisted of surgical wound cleansing with sterile saline solution and antisepsis of the peri-incisional area using 2% alcoholic chlorhexidine. Clinical monitoring was performed every 8 h, during which the following parameters were recorded: spontaneous behavior, posture, respiratory patterns, coat condition, ocular and mucosal appearance, and overall condition.

Pain management was achieved through the intramuscular (IM) administration of Tramadol (2 mg/kg, opioid analgesic) and Dipyrone (25 mg/kg, non-opioid analgesic/antipyretic). Surgical sutures were removed approximately seven days postoperatively.

Postoperative monitoring: During the initial postoperative period, the animals were monitored clinically for general condition, mobility, and apparent abnormalities at the surgical site. The surgical wounds were assessed for visible swelling, erythema, discharge, or other signs of local complication, and general activity and food intake were observed. However, these observations were not recorded using a validated lameness scale or a standardized local-reaction scoring system. Therefore, the postoperative findings are considered descriptive and cannot be used to exclude subtle pain, inflammation, or functional impairment.

### 2.11. Euthanasia and Tissue Harvest

At the conclusion of the experimental periods (30 and 60 days), subjects were transported to the surgical suite for euthanasia. The pre-anesthetic protocol included Midazolam (4 mg/kg) and Acepromazine 0.2% (1 mg/kg) via IM injection. After 30 min, a deep anesthetic plane was induced with Xylazine 2% (5 mg/kg) and Ketamine (35 mg/kg) via IM, followed by an intravenous (IV) bolus of Propofol (30 mg/kg) to ensure cardiorespiratory arrest. Once death was confirmed, the anatomical regions containing the implants were harvested. Carcasses were removed and processed by a specialized biological waste management company.

Experimental sequence: After euthanasia, the bone–implant specimens were harvested and fixed in 10% neutral buffered formalin for two weeks. The fixed specimens were then subjected to reverse-torque testing. Following the mechanical test, the same bone–implant units were processed for histological evaluation. Thus, reverse-torque testing preceded histological processing, and the mechanical procedure may have altered the bone–implant interface and coating surface evaluated histologically. BIC, interface morphology, halo thickness, and coating-delamination findings were therefore interpreted as sequential measurements obtained under this protocol, not as independent measurements of an undisturbed interface.

Formalin fixation before reverse-torque testing may have altered the mechanical properties of bone and the bone–implant interface. Mechanical testing of the same specimens before histology may have also disturbed the interface and coating. Standardization across groups does not eliminate these limitations; it only ensures that the same sequence was applied. Future studies should use separate matched specimens for biomechanical and histological analyses whenever feasible.

Coating delamination was assessed using an ordinal scale from 0 to 3, where 0 indicated extensive delamination and 3 indicated absence of delamination. Higher scores therefore represented better coating integrity. Changes over time were interpreted according to the following direction: a decrease in score indicated greater delamination, whereas an increase indicated less delamination. The temporal and between-group patterns are reported descriptively because the outcome was ordinal, the sample size was limited, and coating condition was confounded with anatomical implant site.

Regional lymph nodes were not harvested or evaluated during necropsy in the present study. Therefore, the study does not provide information regarding lymphatic dissemination, regional lymph node inflammation, macrophage accumulation, particulate transport, or systemic responses to the implant coatings. This omission is acknowledged as a limitation, and no conclusions regarding lymph node or systemic reactions are drawn.

In addition, serial radiographic monitoring during the healing period and post-euthanasia imaging were not performed. Accordingly, no claims are made regarding radiographic bone remodeling, implant migration, radiolucency, or postmortem peri-implant bone changes.

### 2.12. Experimental Design and Bioethics

Each rabbit received all four implant conditions in a split-body arrangement. The rabbit was the independent biological unit, while the four implant sites within each animal were treated as clustered observations. This design reduced between-animal variability but did not make observations within the same rabbit statistically independent. Implant conditions were assigned to predetermined anatomical sites, as described below; therefore, implant conditions and anatomical site were completely confounded.

The split-body arrangement was selected in accordance with the 3Rs principles to reduce the number of animals while maintaining the experimental procedures. Because the implant conditions were not randomized across sites, the design cannot provide an unbiased causal estimate of coating effects independent of laterality or condyle position.

The harvested samples were immersed in 10% buffered formalin in identified containers and transferred for histopathological analysis to the CEDAP Laboratory (Center for Anatomical Pathological Diagnostics), Joinville—SC, Brazil.

### 2.13. Biomechanical Analysis: Reverse-Torque Test (RTQ)

**Figure 5:**
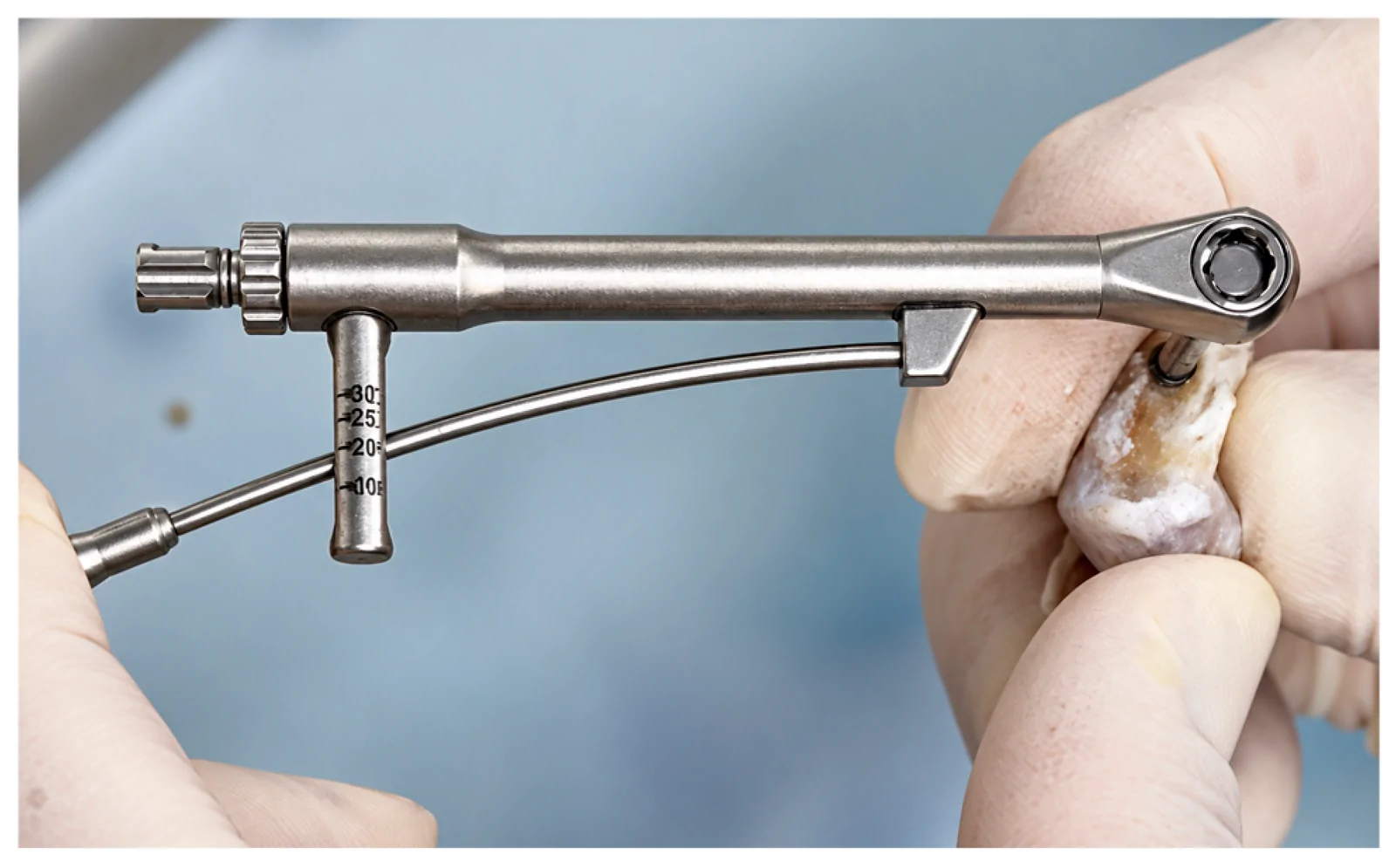
Reverse-torque testing of the bone–implant specimen using a Hiossen Implant mechanical bar-type torque wrench, Torque Wrench—Bar Type, SKU TQWCB.

Following a two-week fixation period in 10% neutral buffered formalin, the biomechanical stability of the bone–implant interface was assessed by reverse-torque testing using a mechanical bar-type torque wrench (Torque Wrench—Bar Type, SKU TQWCB; Hiossen Implant, Ridgefield Park, NJ, USA). The instrument applies torque mechanically by pulling the indicator bar to the selected torque mark and is graduated at 10, 20, and 30 N·cm, with a maximum indicated torque of 30 N·cm, as shown in Figure 5. The torque required to loosen and dislodge each implant was recorded in N·cm. Because formalin fixation may alter the mechanical properties of bone and the bone–implant interface, these measurements reflect the response observed under the study’s preservation protocol and should not be interpreted as representative of the mechanical behavior of a fresh, unfixed interface.

### 2.14. Histopathological and Histochemical Analysis

The specimens were subsequently decalcified using formic acid and processed for histological evaluation. The same bone–implant units that underwent reverse-torque testing were used for histology. Consequently, mechanical testing may have altered the bone–implant interface and coating before BIC, interface morphology, halo thickness, and delamination were assessed. Two pathologists evaluated the sections using predefined criteria; they were not blinded to implant type or experimental period, and discrepancies were resolved by consensus. Formal interobserver agreement was not calculated; therefore, the histological scores are interpreted as semi-quantitative and exploratory.

**Figure 6:**
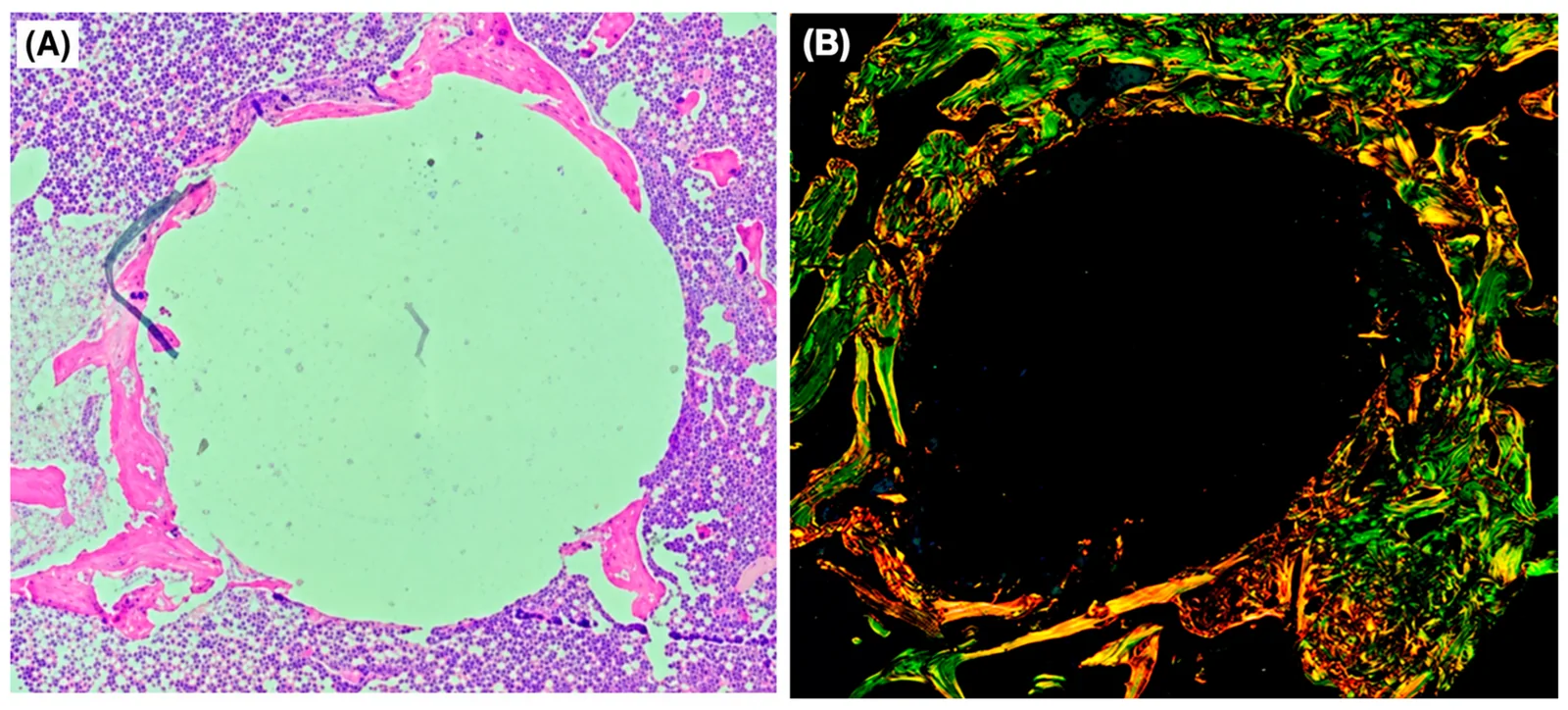
Representative histological sections of peri-implant tissue. (**A**) Hematoxylin and eosin (H&E) staining showing the general morphology of the peri-implant tissue. (**B**) Picrosirius Red staining viewed under polarized light, showing collagen-fiber organization and birefringence. The green, yellow, orange, and red hues represent differences in collagen-fiber birefringence and orientation and should not be interpreted as direct identification of specific collagen types.

Hematoxylin and Eosin (H&E): This technique was employed to visualize the general tissue architecture and organ morphology. Hematoxylin-stained acidic structures (such as nuclei) were stained blue-to-purple, while eosin-stained basic structures (such as the cytoplasm and extracellular matrix) were stained pink-to-red. Under optical microscopy, the samples were evaluated for callus formation, stromal reaction, implant osseointegration, and coating delamination (Figure 6). Blue bars or points represent the 30-day healing period, whereas orange bars or points represent the 60-day healing period.

Picrosirius Red staining was examined by polarized light microscopy to assess the qualitative arrangement and birefringence characteristics of the collagenous matrix surrounding the implants. The observed birefringence patterns were classified according to the original semi-quantitative scoring system. Because polarized-light birefringence is influenced by fiber thickness, alignment, packing, and tissue organization, the color patterns were not considered a direct biochemical or immunohistochemical confirmation of collagen type I or type III. Accordingly, the results were interpreted as optical indicators of collagen-matrix organization and maturation.

Histological outcomes were therefore analyzed as semi-quantitative observations. Because the same specimens underwent reverse-torque testing before histological processing, interface-related findings were interpreted cautiously and were not treated as independent confirmation of the biomechanical results.

### 2.15. Histological Scoring and Semi-Quantitative Analysis

Bone–implant contact was assessed using a semi-quantitative ordinal scale from 0 to 4 rather than by direct measurement of the percentage of implant surface in contact with bone. The score reflected the apparent extent and continuity of bone contact along the interface. It was analyzed and interpreted as an ordinal BIC score, not as a quantitative histomorphometric percentage.

(i) Hematoxylin and Eosin (H&E) Analysis
  - Coating delamination: presence of delamination with or without liberated nanoparticles: 0: extensive; 1: partial; 2: focal; 3: absent.
  - B. Bone Tissue Histological Structure Objective: The objective was to evaluate the peri-implant bone response.
  - (B1) Degree of bone organization: 0: absent; 1: disorganized; 2: partially organized; 3: well-organized.
  - (B2) Presence of osteoid: 0: absent; 1: present.
  - (B3) Osteoclastic proliferation: 0: absent; 1: discrete; 2: moderate; 3: marked.
  - (B4) Bone-to-implant contact (BIC): 0: none; 1: focal (<25%); 2: partial (25–50%); 3: extensive (50–75%); 4: >75%.
  - (B5) Peri-implant interface thickness: halo measurement within the region of interest (ROI) in millimeters (mm).
  - BIC refers to an ordinal histological estimate of the extent of bone contact at the interface in this study. Direct percentage measurements were not calculated; therefore, the outcome is reported as a semi-quantitative BIC score.
  - C. Cartilaginous Proliferation and Healing Objective: The objective was to identify endochondral or fibrous repair pathways.
  - (C1) Predominant repair tissue: 1: fibrous; 2: cartilaginous; 3: osseous.
  - (C2) Repair tissue maturation: 0: absent; 1: immature; 2: partial; 3: mature.
  - D. Vascular Response Objective: The objective was to evaluate neovascularization or microvascular alterations.
  - Neovascularization: 0: absent; 1: mild; 2: moderate; 3: intense.
  - E. Inflammatory Response Objective: The objective was to assess immunological response or material rejection.
  - (E1) Acute inflammatory infiltrate: 0: absent; 1: present.
  - (E2) Chronic inflammatory infiltrate: 0: absent; 1: mild; 2: moderate; 3: intense.
  - (E3) Presence of multinucleated giant cells: 0: absent; 1: present.
  - F. Bone Neoformation Objective: The objective was to verify osteoinduction and osteoconduction.
  - (F1) Maturity of newly formed bone: 1: immature (osteoid); 2: mixed; 3: mature.
(ii) Picrosirius Red Staining Analysis
  - G. Picrosirius Red Birefringence Pattern. Objective: The objective was to evaluate collagen fiber organization under polarized-light microscopy. Orange-red and green birefringence were recorded as optical patterns and were not interpreted as molecular identification or quantification of collagen types I and III.
  - (G1) Type I collagen: 0: absent; 1: mild; 2: moderate; 3: intense.
  - (G2) Type III collagen: 0: absent; 1: mild; 2: moderate; 3: intense.
  - (G3) Collagen ratio: 1: type I < type III; 2: type I = type III; 3: type I > type III.

### 2.16. Statistical Analysis

The rabbit was the independent biological unit, and the four implant sites within each rabbit were treated as clustered observations. Reverse torque (RTQ) was analyzed using a linear mixed-effects model with the implant-site condition, healing period, and their interaction as fixed effects and the rabbit as a random intercept. Because each surface condition was permanently assigned to a specific anatomical site, the condition term represents a combined implant-site condition and cannot be interpreted as an independent coating effect. Model estimates are therefore exploratory. Ordinal histological outcomes were summarized descriptively using individual scores and group-level distributions; no implant-level inferential *p*-values were used for these outcomes because of the small animal-level sample and fixed treatment-to-site allocation. For the RTQ mixed model, two-sided *p* < 0.05 was used as an exploratory significance threshold.

### 2.17. Data Presentation

Continuous outcomes were summarized using the mean and standard deviation or the median and interquartile range, as appropriate. Histological outcomes were summarized as ordinal scores with individual observations and outcome-specific sample sizes when available. The rabbit remained the independent biological unit, whereas implants and implant sites within each animal were treated as clustered observations. Group-level summaries are presented for each condition and healing period.

## 3. Results

### 3.1. Experimental Design and Animal Models

Twelve rabbits were initially enrolled, with six allocated to each healing period. Two animals were lost before endpoint and were not replaced, leaving ten analyzed rabbits: five at 30 days and five at 60 days. Each analyzed rabbit contributed four implant-site observations. The final animal-level sample size was *n* = 5 for each healing period, and the four observations within each rabbit were treated as clustered observations. The study did not include a priori power calculation. Group-level summaries are presented for each condition and healing period. Where available, individual observations and dispersion measures are reported. Ordinal outcomes are interpreted as semi-quantitative scores rather than continuous measurements.

All rabbits were males aged between 6 and 12 months, with a mean weight of 3.21 kg (range: 2.8 to 3.5 kg). The screws were numbered for statistical analysis and corresponded to the following groups:

- Implant/screw P1 = left knee, lateral condyle = LL = niobium gel;
- Implant/screw P2 = right knee, lateral condyle = RL = uncoated;
- Implant/screw P3 = right knee, medial condyle = RM = Graphene-DLC;
- Implant/screw P4 = left knee, medial condyle = LM = niobium CVD.

Analyses were performed based on the type of implant/screw (1, 2, 3, and 4) and the implantation period in the condyles (30 and 60 days).

Implant conditions were assigned to a predetermined anatomical site in every animal. Consequently, coating condition and anatomical position were completely confounded, and the effects of laterality and medial versus lateral condyle cannot be separated from the effects associated with the surface condition. The present results therefore describe the performance of the combined implant-site conditions under this protocol and do not provide an unbiased causal estimate of coating effects. Randomized or counterbalanced allocation of implant conditions across sites is required in future experiments.

### 3.2. Reverse-Torque Test—RTQ

Implant stability was assessed using the reverse-torque test (RTQ). After two weeks of fixation in 10% neutral buffered formalin, the fixed bone–implant specimens underwent reverse-torque testing. The torque required to unscrew and dislodge each implant was measured using a calibrated analog torque meter and recorded in N.cm. The same specimens were subsequently processed for histological evaluation. Individual values and descriptive summaries are presented in Table 1.

Mean reverse-torque values were higher in the 60-day cohort than those in the 30-day cohort for all four implant-site conditions. At 30 days, the mean ± SD values were 3.46 ± 0.58 N.cm for P1, 2.48 ± 0.66 N.cm for P2, 2.98 ± 1.04 N.cm for P3, and 3.22 ± 0.88 N.cm for P4, and at 60 days, the corresponding values were 4.08 ± 1.82, 5.14 ± 1.26, 5.14 ± 1.26, and 4.08 ± 1.82 N.cm, respectively. In the exploratory linear mixed-effects model, the condition-by-period interaction was positive for P2 versus P1 (estimate 2.04 N.cm, 95% CI 0.57 to 3.51; *p* = 0.006) and for P3 versus P1 (estimate 1.54 N.cm, 95% CI 0.07 to 3.01; *p* = 0.039), whereas the P4-versus-P1 interaction was not statistically significant (estimate 0.24 N.cm; *p* = 0.748). The main 60-day period coefficient for the P1 reference condition was not significant (estimate 0.62 N.cm; *p* = 0.432). Because surface condition and anatomical site were completely confounded, these model coefficients describe differences among combined implant-site conditions and do not establish coating-specific causal effects.

### 3.3. Laboratory Measurements

#### 3.3.1. Coating Delamination

**Figure 7:**
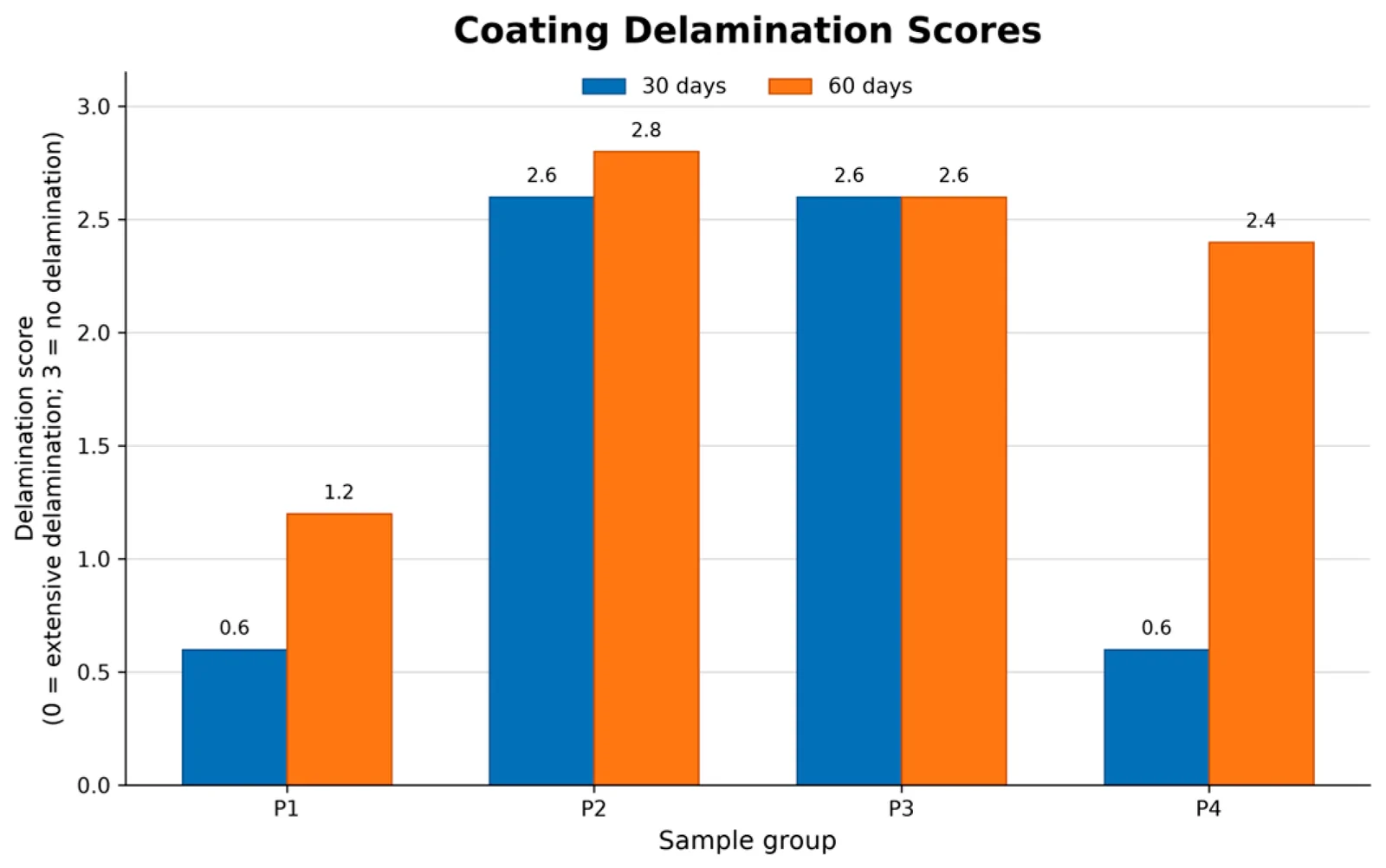
Coating-delamination scores according to implant-site condition and healing period. A score of 0 indicates extensive delamination and a score of 3 indicates absence of delamination; higher scores indicate better coating integrity. Values are descriptive group summaries for *n* = 5 rabbits per condition and period. Blue bars/points represent the 30-day healing period, and orange bars/points represent the 60-day healing period.

Coating delamination was evaluated using a four-point ordinal scale: 0 indicated extensive delamination, 1 partial delamination, 2 focal delamination, and 3 absence of delamination (Figure 7). Higher scores therefore indicated better coating integrity.

The uncoated titanium and Graphene-DLC conditions showed higher reported integrity scores than the niobium-containing conditions under the study protocol. These differences are descriptive and do not provide definitive evidence of coating adhesion, particle release, or a causal effect of surface chemistry. Reverse-torque and histological assessment were performed sequentially on the same fixed specimens, which further limits interpretation.

#### 3.3.2. Histological Structure of Bone Tissue

Histological observations of peri-implant bone were recorded using predefined semi-quantitative ordinal scores. The present study did not directly measure the molecular pathways, mineralization kinetics, corrosion resistance, biofilm formation, or osteogenic mechanisms that may be discussed in the literature. Such mechanisms are therefore hypotheses rather than findings demonstrated by this experiment.

The biological mechanisms underlying the observed histological and biomechanical findings remain uncertain because molecular and biochemical pathways were not directly assessed in the present study. Mechanisms involving FAK/p38 signaling, TNF-α, IL-1β, RANK/RANKL activation, calcium–phosphate nucleation, and macrophage-mediated responses may provide plausible explanations based on previous studies of carbon-based and metal-oxide surfaces; however, these pathways were not measured by gene expression, protein, immunohistochemical, or biochemical assays in the present experiment. Accordingly, they should be considered hypotheses for future investigation rather than mechanisms demonstrated by the current data.

The present study did not directly measure mineralization kinetics, corrosion resistance, or biofilm formation. Therefore, the tested surfaces should not be described as producing accelerated mineralization, exhibiting high corrosion resistance, or possessing anti-biofilm activity based on the present results alone. Such properties may be biologically plausible or may have been reported for related materials in previous studies, but their presence and magnitude in the surfaces evaluated here require direct confirmation using dedicated mineralization, electrochemical corrosion, and microbiological assays.

#### 3.3.3. Degree of Bone Organization

**Figure 8:**
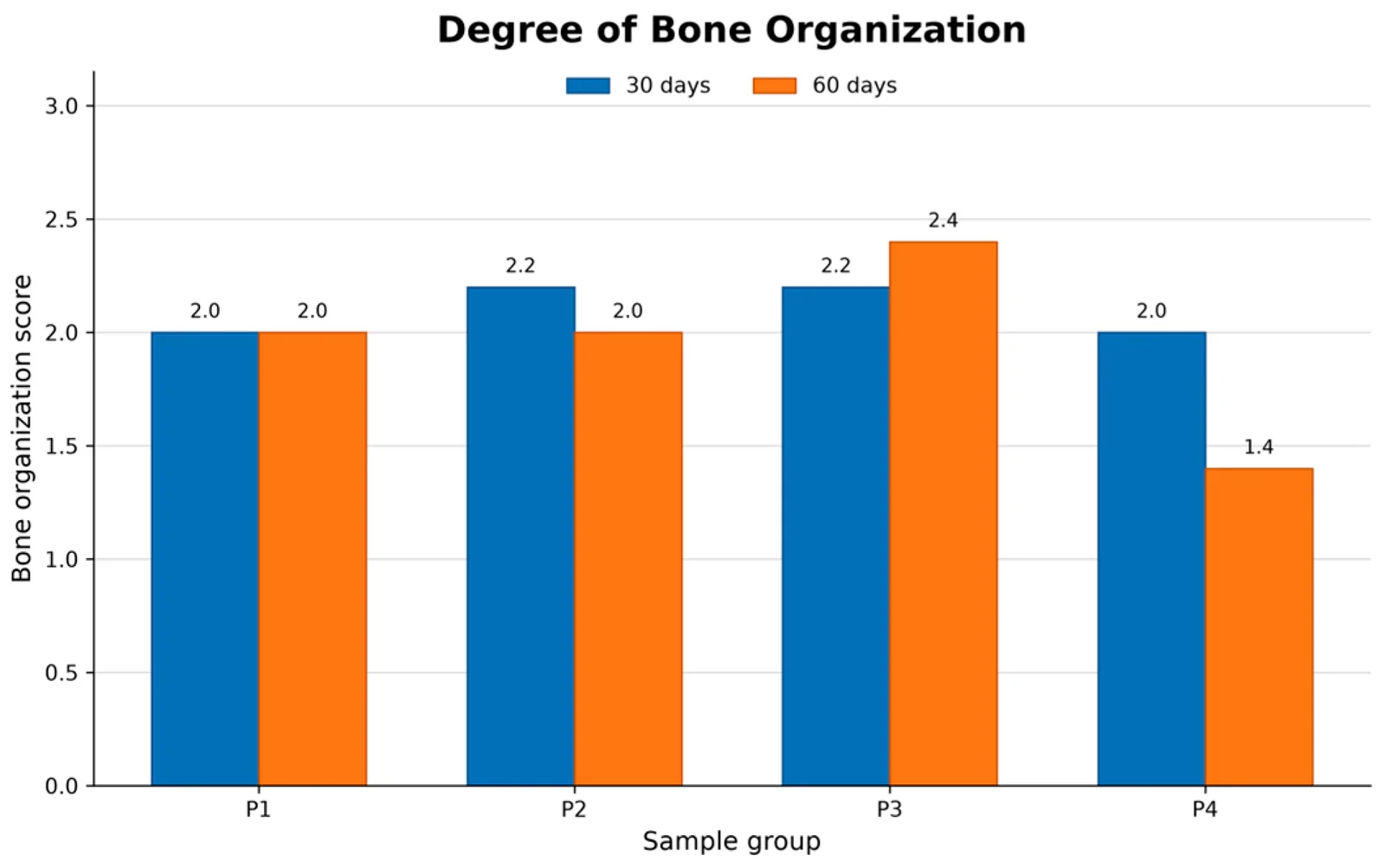
Degrees of bone organization at 30 and 60 days. The outcome is an ordinal histological score and is presented as descriptive group summaries for *n* = 5 rabbits per condition and period. Blue bars/points represent the 30-day healing period, and orange bars/points represent the 60-day healing period.

Bone organization was assessed using an ordinal histological score (Figure 8). The scores showed group- and period-specific variation rather than a uniform progression across all implant-site conditions. The results do not demonstrate a uniform progression toward lamellar bone or a coating-specific osteoinductive effect.

The plotted values are descriptive group summaries. The observed differences do not demonstrate a uniform maturation trajectory, and the data do not establish that coating delamination, particulate debris, or a specific mechanical-signaling pathway caused the observed bone organization pattern.

#### 3.3.4. Presence of Osteoid

**Figure 9:**
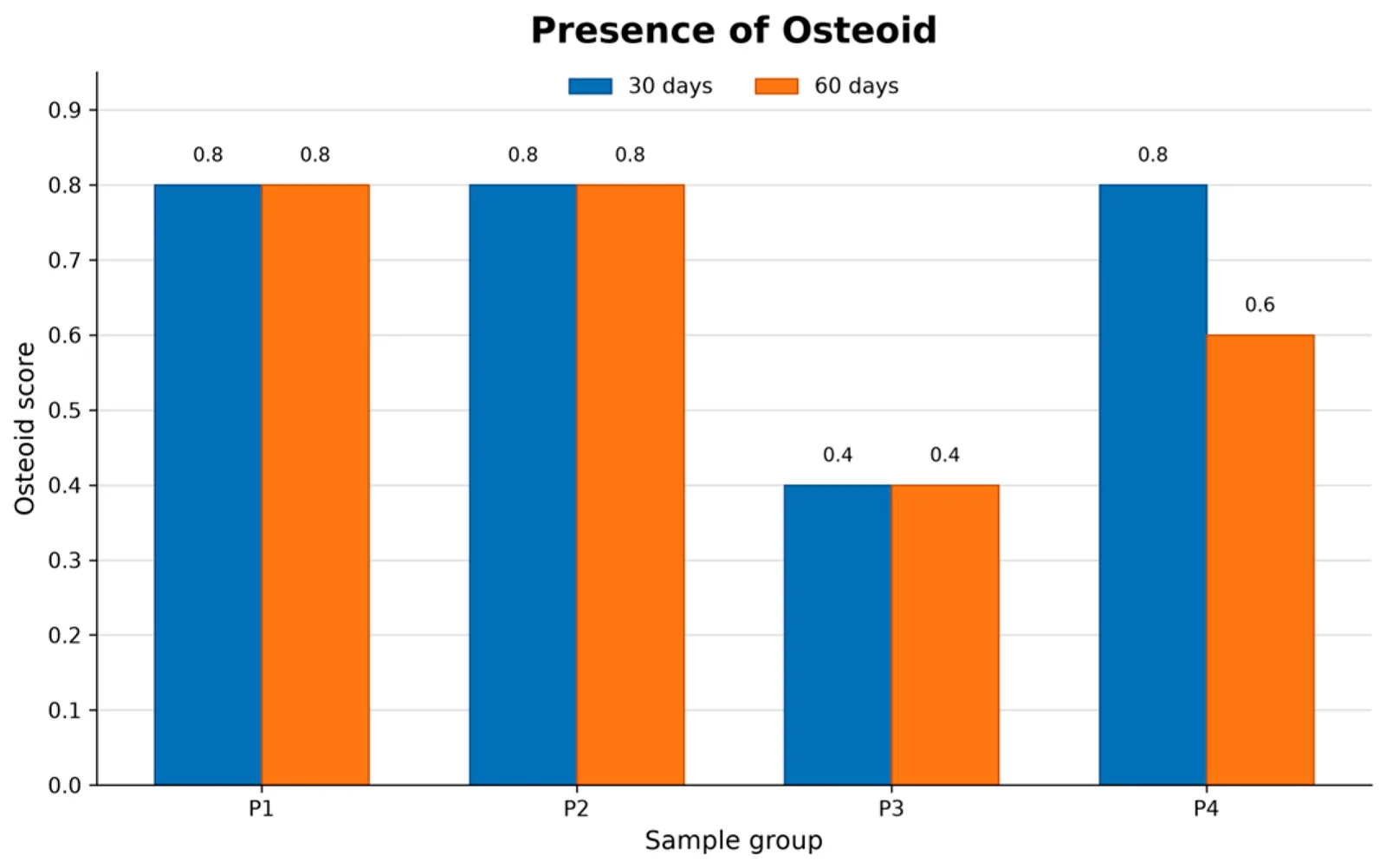
Presence of osteoid at 30 and 60 days. Blue bars/points represent the 30-day healing period, and orange bars/points represent the 60-day healing period.

The presence of osteoid was assessed using a semi-quantitative ordinal score (Figure 9).

The lower descriptive score observed for P3 should not be interpreted as evidence of impaired matrix deposition or accelerated mineralization; osteoid volume, mineralization kinetics, calcium–phosphate nucleation, and osteoblast activity were not directly measured. The data do not demonstrate that the Graphene-DLC condition shortened the interval between osteoid formation and mineralization or promoted calcium–phosphate nucleation.

#### 3.3.5. Osteoclastic Proliferation

**Figure 10:**
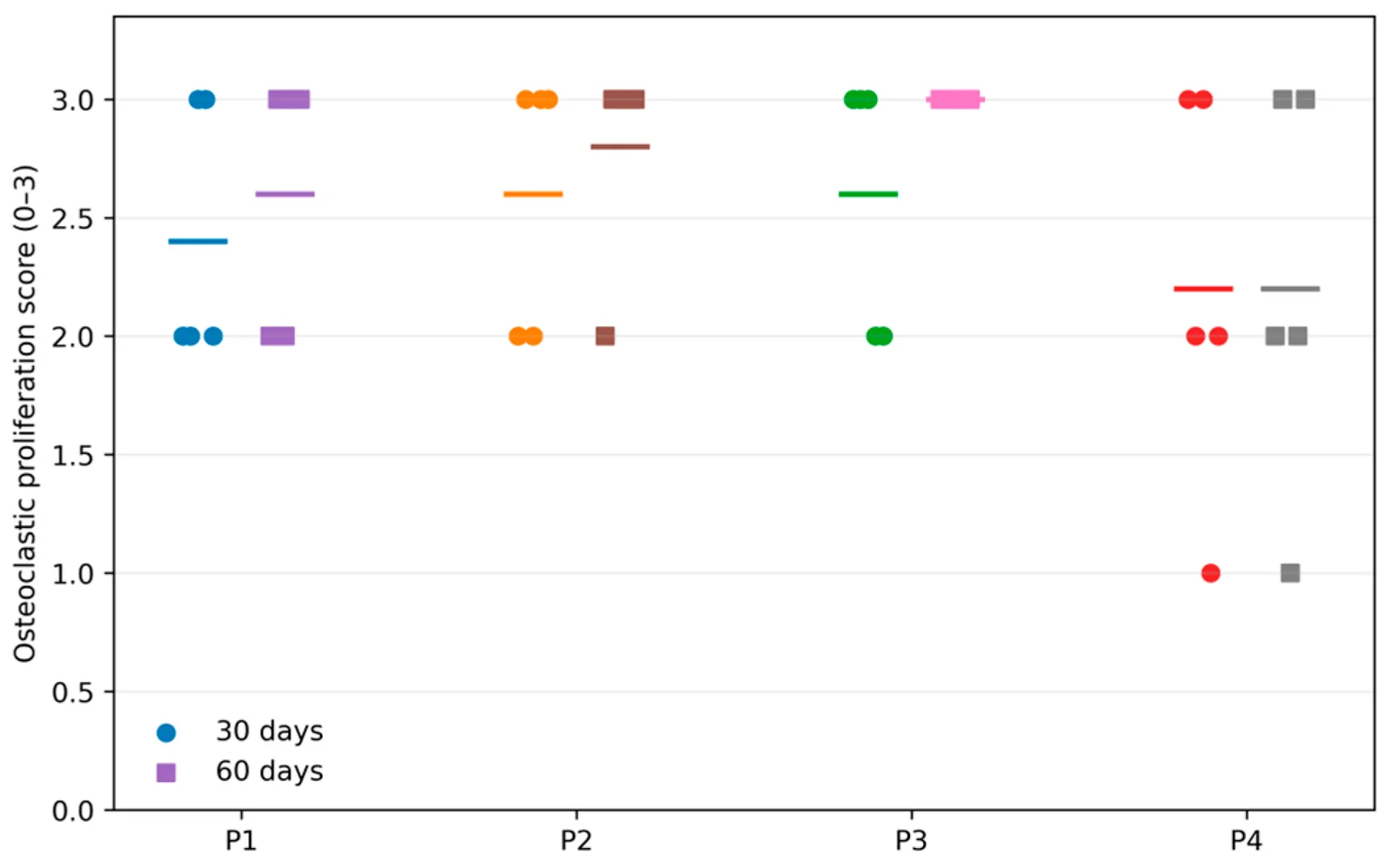
Semi-quantitative osteoclastic-proliferation scores at 30 and 60 days (*n* = 5 rabbits per condition and period). Points represent individual ordinal scores; horizontal markers indicate descriptive group means. Blue bars/points represent the 30-day healing period, and orange bars/points represent the 60-day healing period.

Osteoclastic proliferation was scored on an ordinal scale from 0 (absent) to 3 (marked). Mean scores at 30 and 60 days, respectively, were 2.40 and 2.60 for P1, 2.60 and 2.80 for P2, 2.60 and 3.00 for P3, and 2.20 and 2.20 for P4 (Figure 10). These values showed modest variation among conditions and healing periods without a uniform temporal pattern.

The present data does not support a definitive statement that the uncoated titanium or Graphene-DLC conditions had lower osteoclastic proliferation. Similarly, the niobium-containing scores do not establish pathological bone resorption, particulate debris release, RANK/RANKL activation, localized osteolysis, or a coating-specific inflammatory mechanism.

#### 3.3.6. Bone-to-Implant Contact

**Figure 11:**
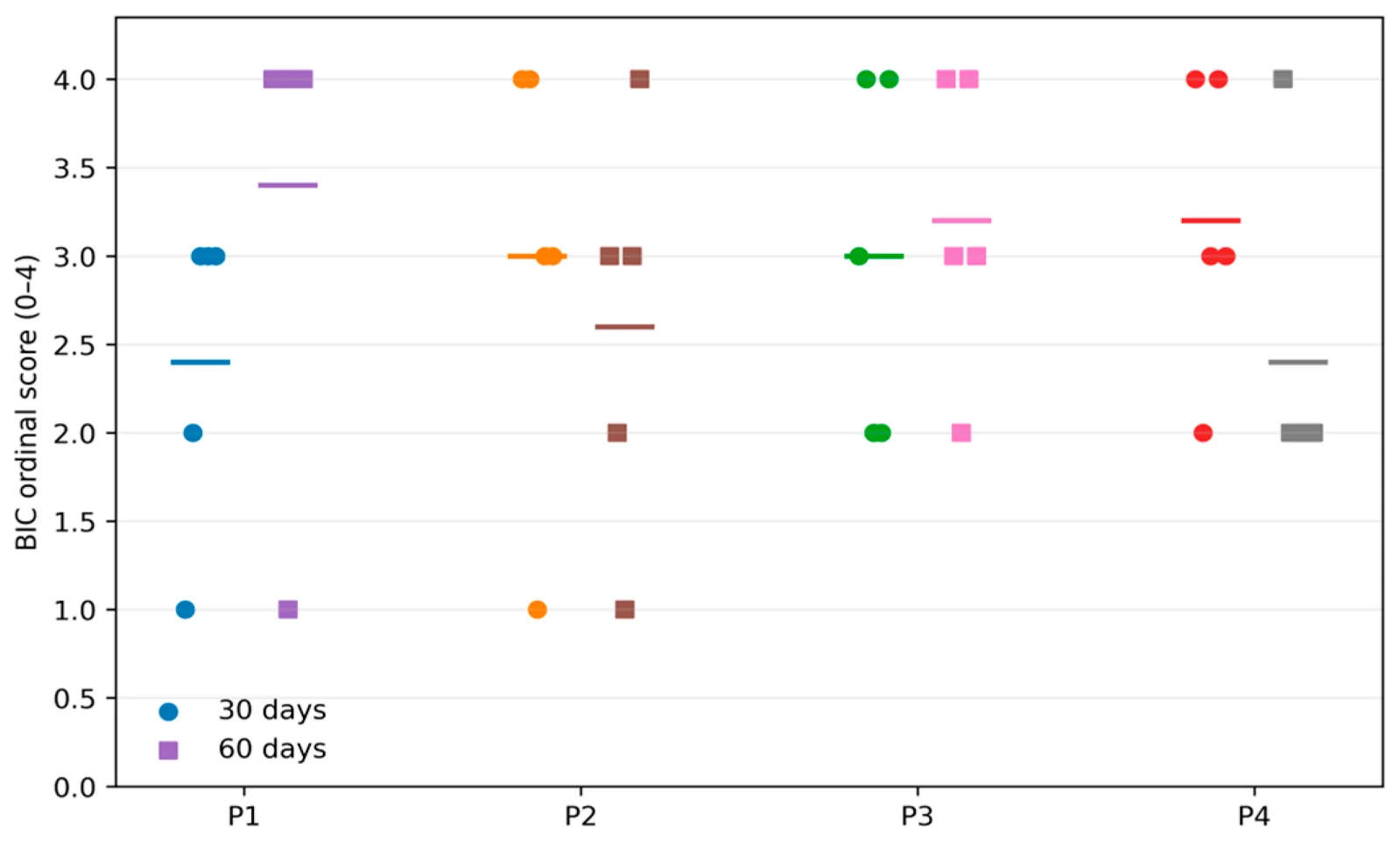
Semi-quantitative bone-to-implant contact (BIC) scores at 30 and 60 days (*n* = 5 rabbits per condition and period). Points represent individual ordinal scores; horizontal markers indicate descriptive group means. Blue bars/points represent the 30-day healing period, and orange bars/points represent the 60-day healing period.

Bone-to-implant contact (BIC) was assessed using a semi-quantitative ordinal score ranging from 0 to 4. Mean scores at 30 and 60 days, respectively, were 2.40 and 3.40 for P1, 3.00 and 2.60 for P2, 3.00 and 3.20 for P3, and 3.20 and 2.40 for P4 (Figure 11). Individual scores ranged from 1 to 4. The direction of change varied among implant-site conditions, with no uniform temporal increase. BIC is reported as a semi-quantitative ordinal score rather than as a direct percentage histomorphometric measurement. No conclusion regarding coating superiority, osteoclastic coupling, or secondary stability is drawn from a descriptive score comparison alone.

BIC was not formally interpreted as a biological mechanism or as evidence of a coating-specific effect. The present data does not establish that the coexistence of BIC and osteoclastic scores represents healthy coupling, accelerated remodeling, or superior interfacial performance. The present study did not directly measure wettability, cellular anchoring, osteogenesis, mineralization, or mechanically guided remodeling. These mechanisms may be discussed as hypotheses based on the previous literature but are not demonstrated by the current data.

#### 3.3.7. Thickness of Bone–Implant Interface–Peri-Implant Halo (ROI)

**Figure 12:**
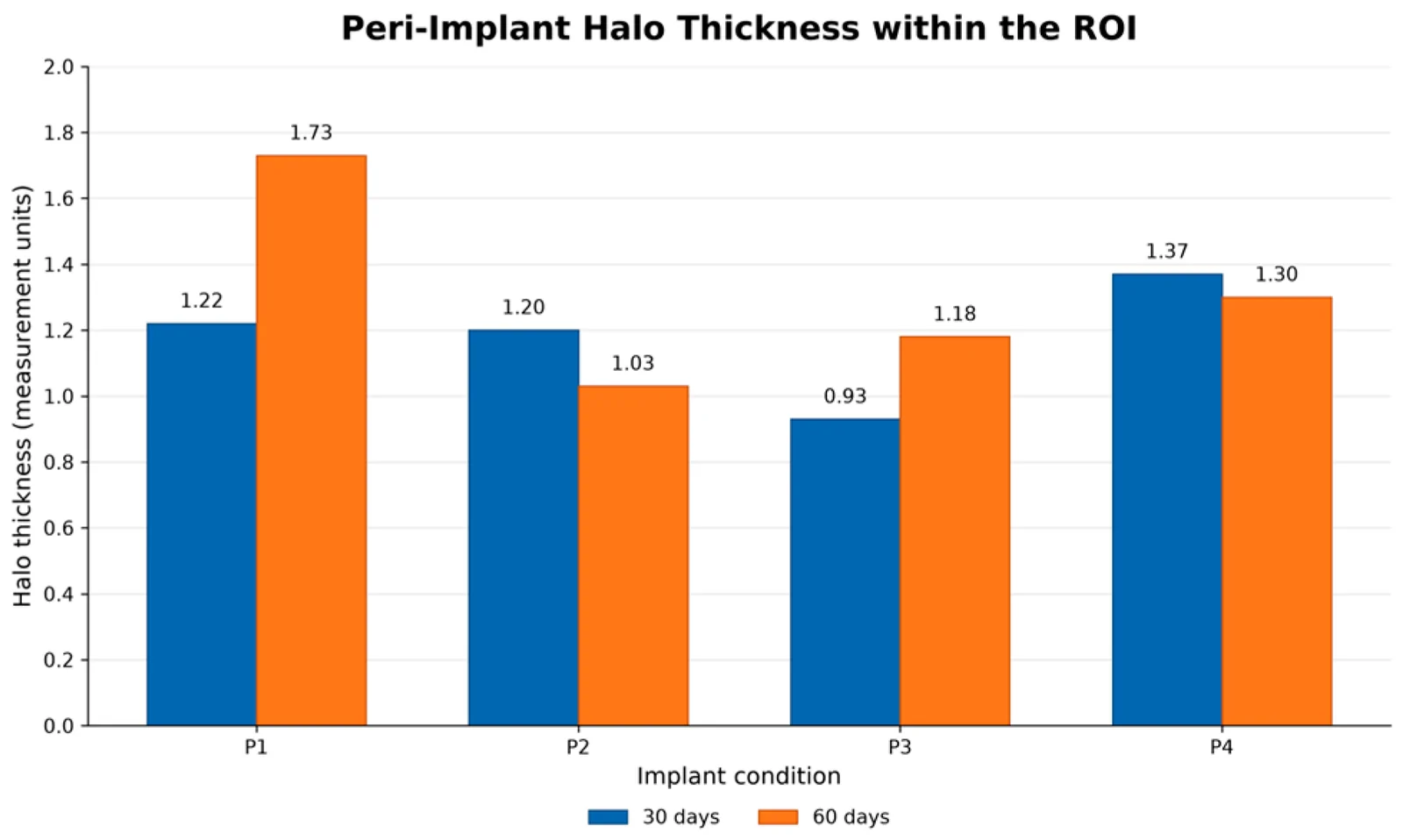
Peri-implant halo thickness within region of interest (ROI) according to implant condition and healing period. Blue bars/points represent the 30-day healing period, and orange bars/points represent the 60-day healing period.

Peri-implant interface thickness was recorded in millimeters as the halo measurement within the region of interest (Figure 12).

The graph presents the reported peri-implant halo measurements at 30 and 60 days. The values do not establish a fibrous capsule, mechanical instability, foreign-body reaction, particulate release, or impaired bone anchorage. The relationship between halo thickness, coating integrity, inflammatory response, and reverse torque was not formally established.

#### 3.3.8. Predominant Type of Scar Tissue

**Figure 13:**
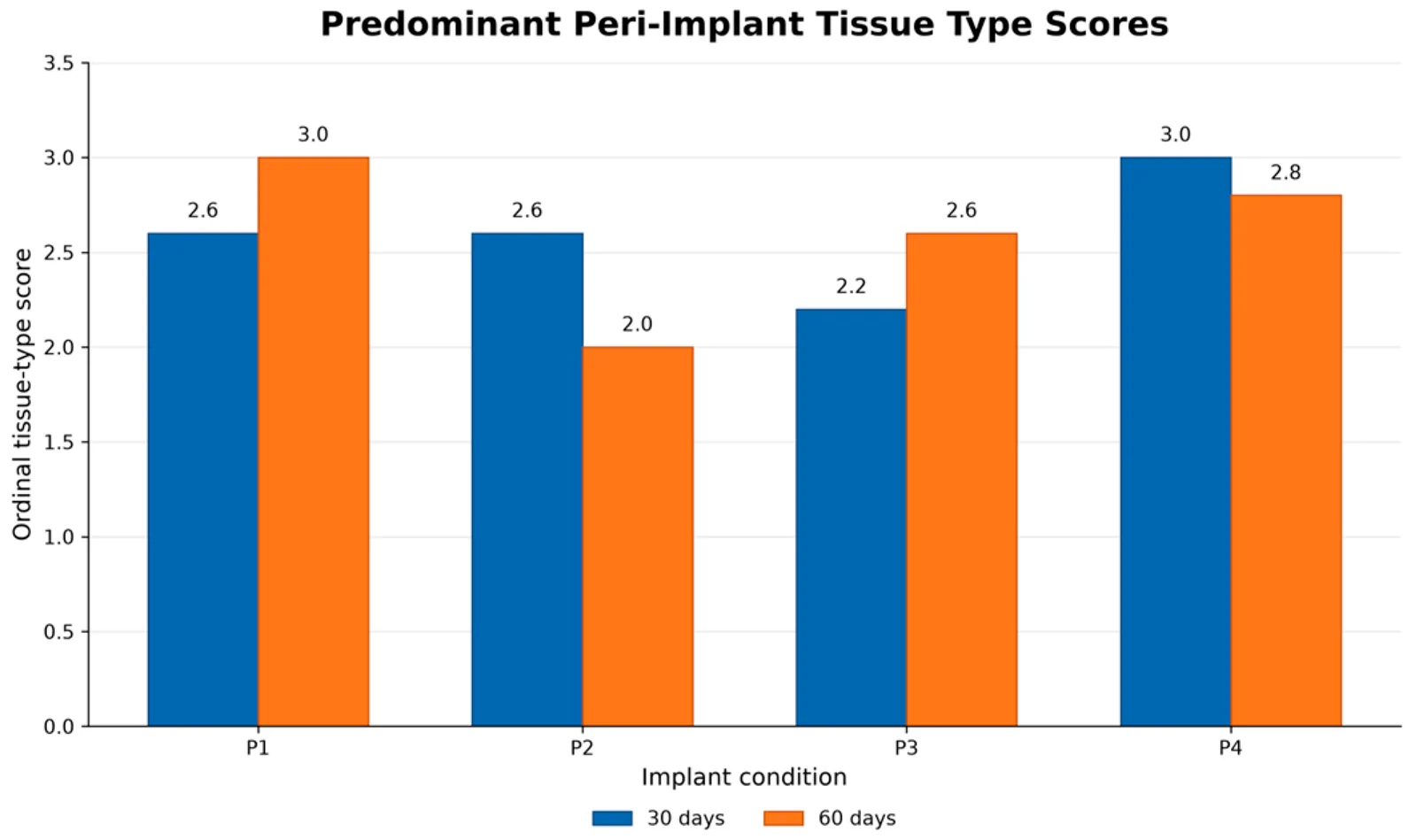
Predominant peri-implant tissue-type scores according to implant condition and healing period. Blue bars/points represent the 30-day healing period, and orange bars/points represent the 60-day healing period.

The histological evaluation of the predominant type of scar tissue (Figure 13) surrounding the peri-implant interface revealed critical variations in tissue quality and composition among the groups.

The graph presents the reported ordinal tissue-type scores. The score categories and their definitions are specified in the histological scoring protocol. These scores do not directly measure coating delamination, inflammatory cytokines, macrophage activation, foreign-body reaction, osteoblast differentiation, or osteoconductivity.

Observed tissue-type patterns do not establish that niobium delamination caused fibrous encapsulation, macrophage activation, reactive oxygen species release, cytokine production, or impaired osteoblast differentiation. These mechanisms were not directly measured. The present histological observations cannot determine whether particulate debris or a chronic foreign-body reaction was present because particles, macrophage activation, and molecular inflammatory markers were not specifically characterized.

#### 3.3.9. Maturation of Repair Tissue

**Figure 14:**
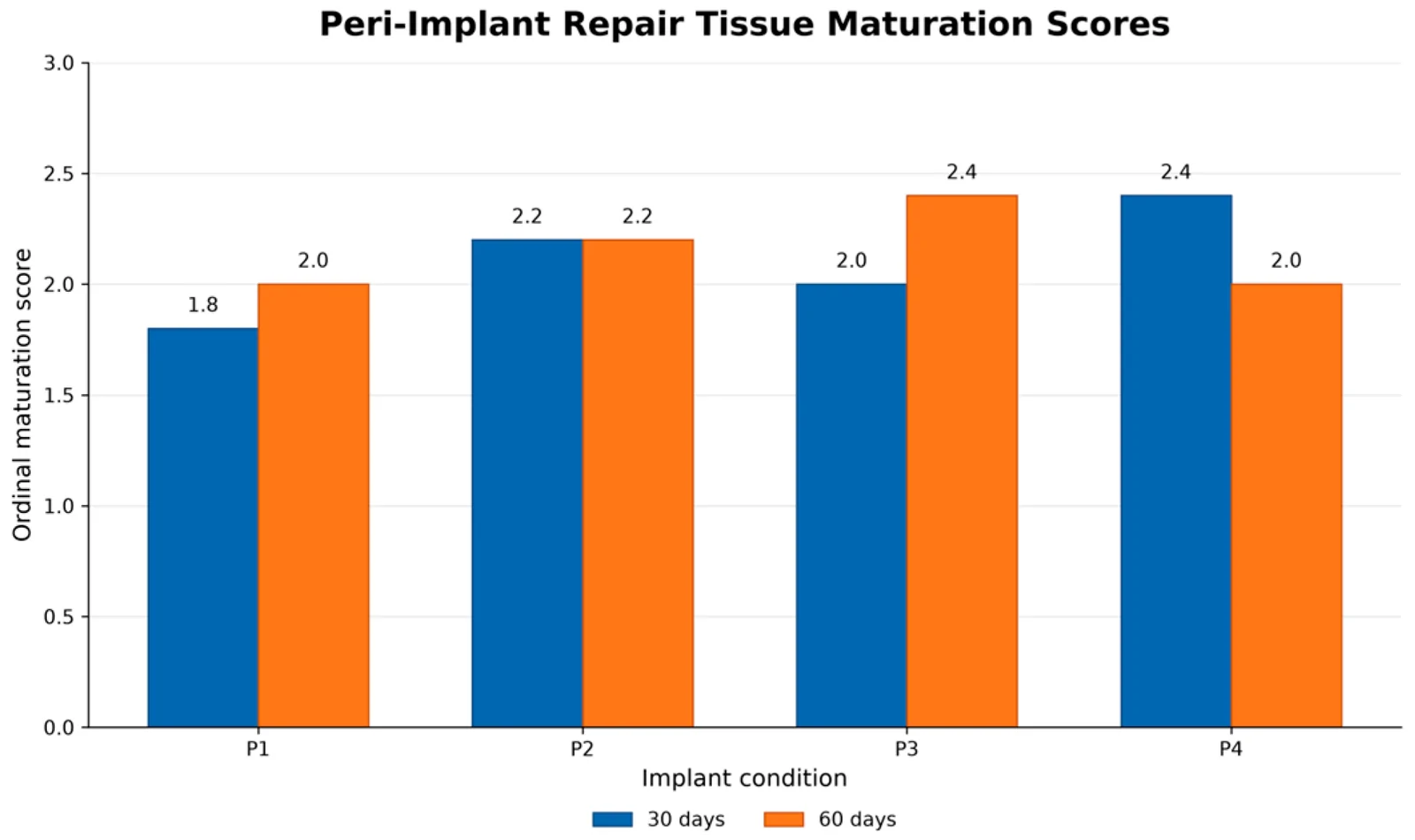
Peri-implant repair tissue maturation scores according to implant condition and healing period. Blue bars/points represent the 30-day healing period, and orange bars/points represent the 60-day healing period.

Repair tissue maturation was evaluated using an ordinal histological score (Figure 14). Scores varied among implant-site conditions and healing periods without a uniform temporal pattern. No coating-specific advantage or disadvantage is inferred from these descriptive values.

The values showed variation among implant-site conditions and periods. These patterns do not demonstrate that the Nb-CVD process accelerated early adhesion, protein adsorption, or mineralization. Likewise, the descriptive P3 pattern does not demonstrate delayed or progressive osteogenic maturation, a specific carbon surface kinetic, or superior lamellar bone formation.

#### 3.3.10. Vascular Response–Neovascularization

**Figure 15:**
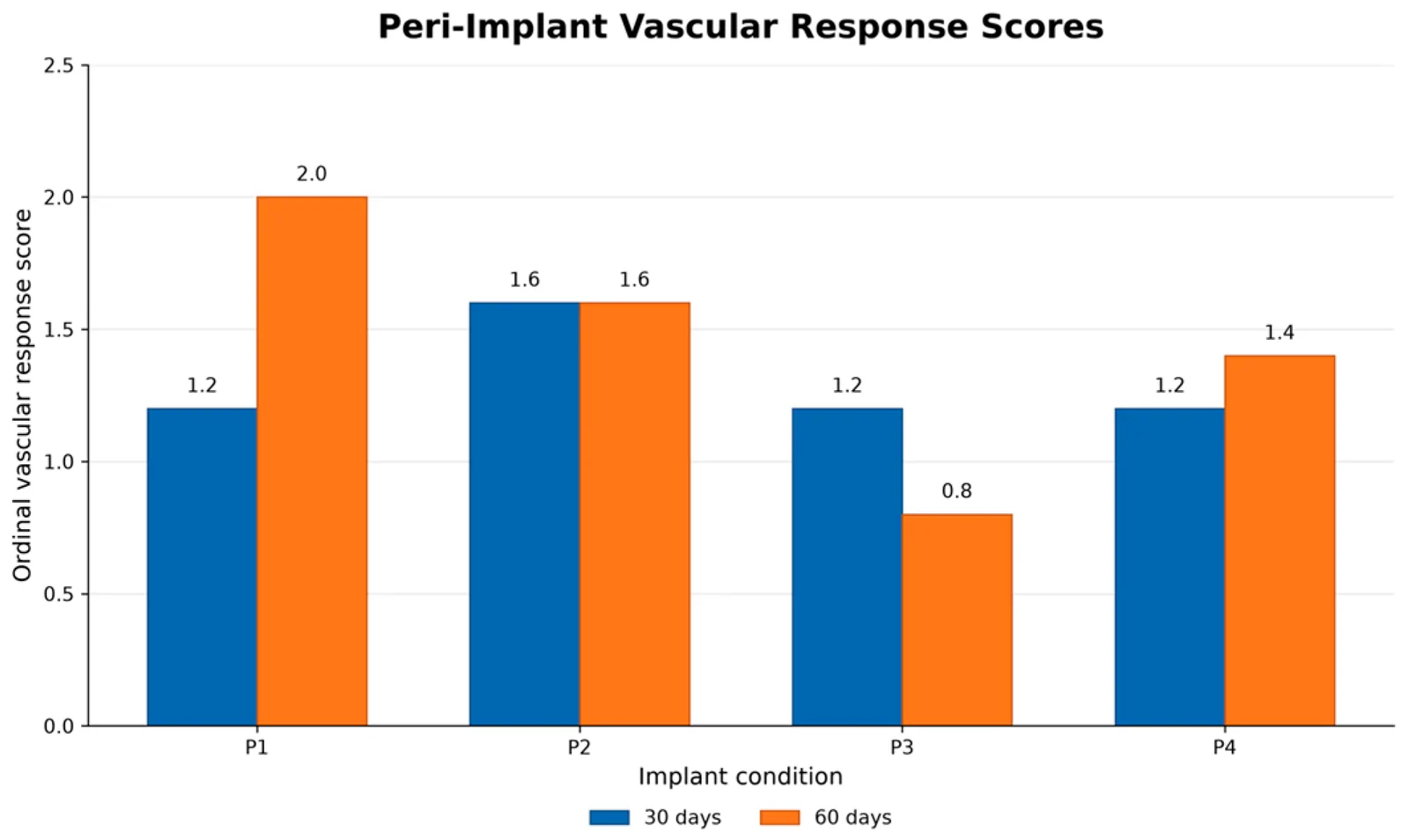
Peri-implant vascular response scores at 30 and 60 days. Blue bars/points represent the 30-day healing period, and orange bars/points represent the 60-day healing period.

The peri-implant vascular response was evaluated using an ordinal histological scoring system. Condition- and period-specific variation is observed in Figure 15.

The observed scores describe vascular findings under the evaluated conditions and do not establish equivalence, absence of cytotoxicity, ischemia, improved biocompatibility, or a specific angiogenic mechanism.

#### 3.3.11. Inflammatory Response

The tissue response to implant placement begins with vascular injury and local bleeding, which initiate hemostasis and the subsequent inflammatory and reparative phases of wound healing. Characterization of the inflammatory response surrounding metallic implants is important for evaluating local biocompatibility and identifying potentially adverse tissue reactions.

A transient acute inflammatory infiltrate may reflect the normal response to surgical trauma and implant insertion. In contrast, persistent or moderate-to-severe inflammatory infiltration during later stages of healing may indicate delayed tissue repair, local irritation, or an adverse host response. The assessment of macrophages and multinucleated giant cells is also relevant because these cells may participate in the tissue response to implanted materials and to particulate debris. Accordingly, the local inflammatory response was evaluated using a predefined histological scoring system consistent with the principles described in ISO 10993-6 (Biological evaluation of medical devices—Part 6: Tests for local effects after implantation. International Organization for Standardization: Geneva, Switzerland, 2016) [16] for the biological evaluation of medical devices after implantation.

However, histological inflammation scores alone cannot establish implant rejection, cytotoxicity, or a specific mechanism of tissue injury. Such conclusions require correlation with clinical observations, implant stability, coating integrity, particle characterization, and, when appropriate, molecular or microbiological analyses.

#### 3.3.12. Acute Inflammatory Infiltrate

**Figure 16:**
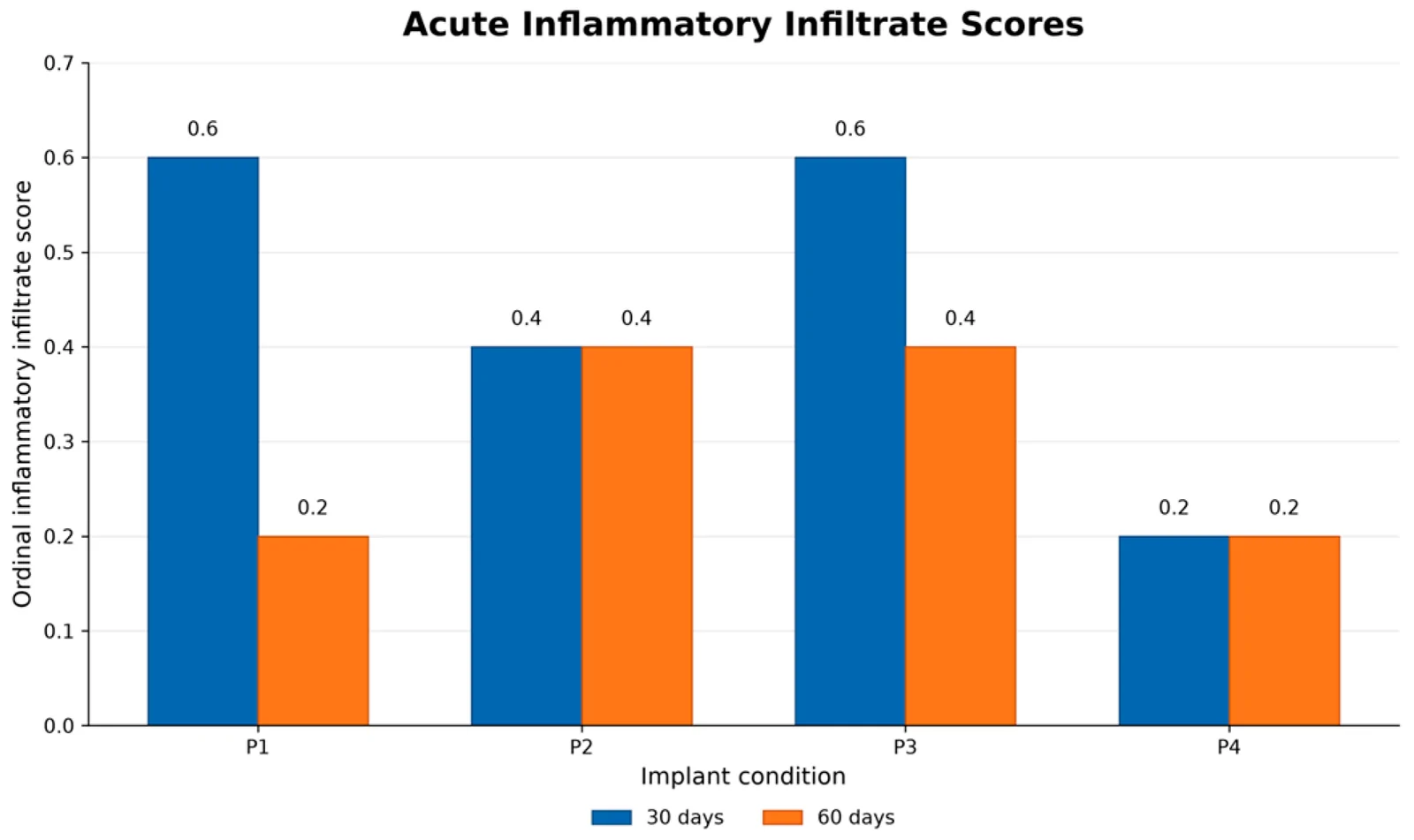
Acute inflammatory-infiltrate scores according to implant condition and healing period. Blue bars/points represent the 30-day healing period, and orange bars/points represent the 60-day healing period.

Acute inflammatory-infiltrate scores were higher at 30 days than at 60 days for P1 and P3, whereas P2 and P4 showed similar reported scores at both healing periods (Figure 16). An acute inflammatory infiltrate may reflect the local tissue response to surgical trauma and implant placement. However, the present ordinal scores do not directly establish neutrophil activation, cytokine production, reactive oxygen species release, tissue injury, infection, or implant rejection. These mechanisms were not directly measured in this study. Therefore, the results are interpreted as differences in histological inflammatory-infiltrate scores under the conditions of the experiment.

#### 3.3.13. Chronic Inflammatory Infiltrate

**Figure 17:**
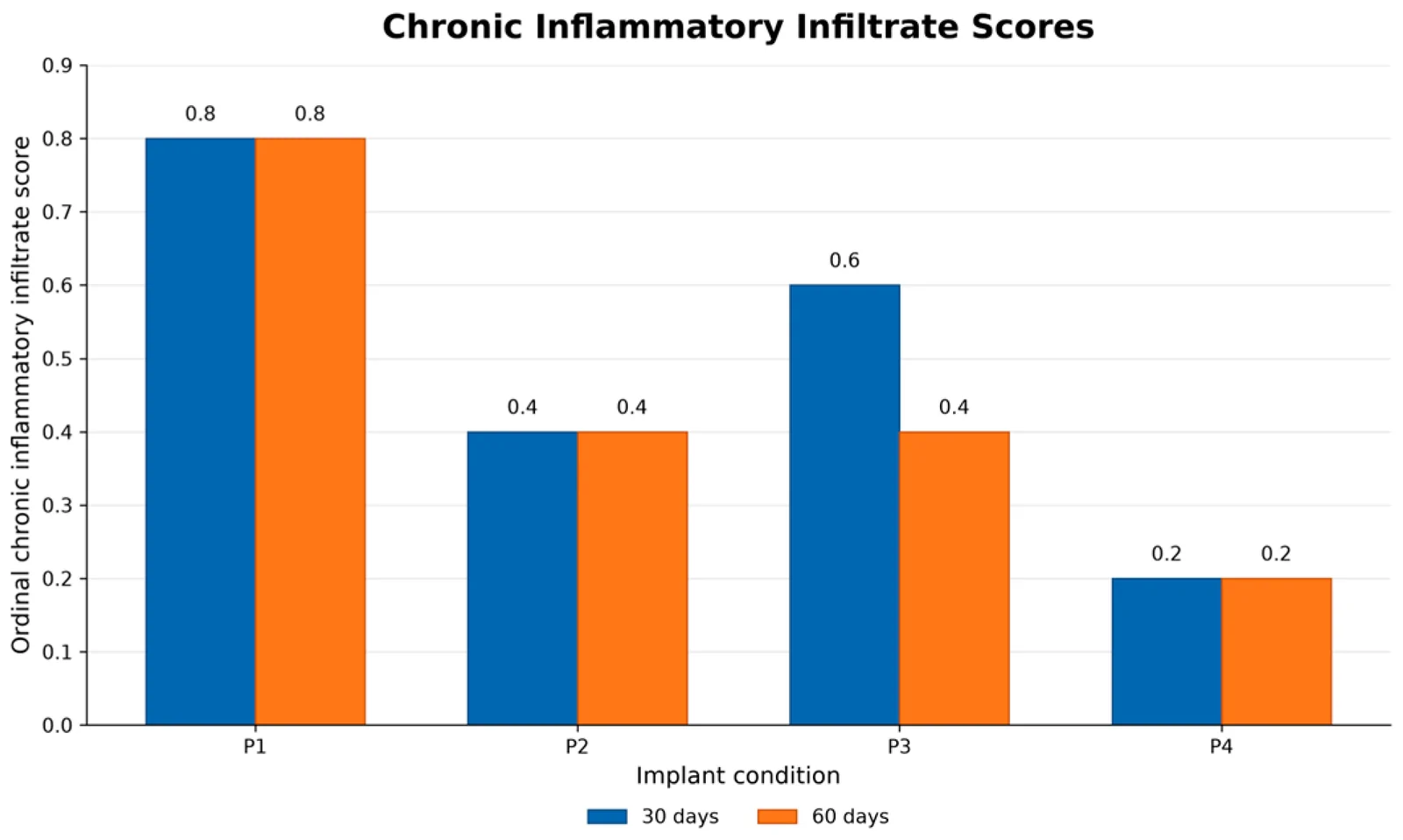
Chronic inflammatory infiltrate scores according to implant condition and healing period. Blue bars/points represent the 30-day healing period, and orange bars/points represent the 60-day healing period.

The chronic inflammatory response was evaluated using an ordinal histological scoring system. The reported scores were 0.8/0.8 for P1, 0.4/0.4 for P2, 0.6/0.4 for P3, and 0.2/0.2 for P4 at 30 and 60 days, respectively (Figure 17). The observed scores were generally low, particularly for P2, P3, and P4. However, low ordinal scores alone do not establish the absence of a foreign-body reaction, progressive fibrosis, persistent inflammation, or systemic compatibility; these outcomes were not directly measured using molecular, microbiological, or systemic assays.

#### 3.3.14. Presence of Multinucleated Giant Macrophages

**Figure 18:**
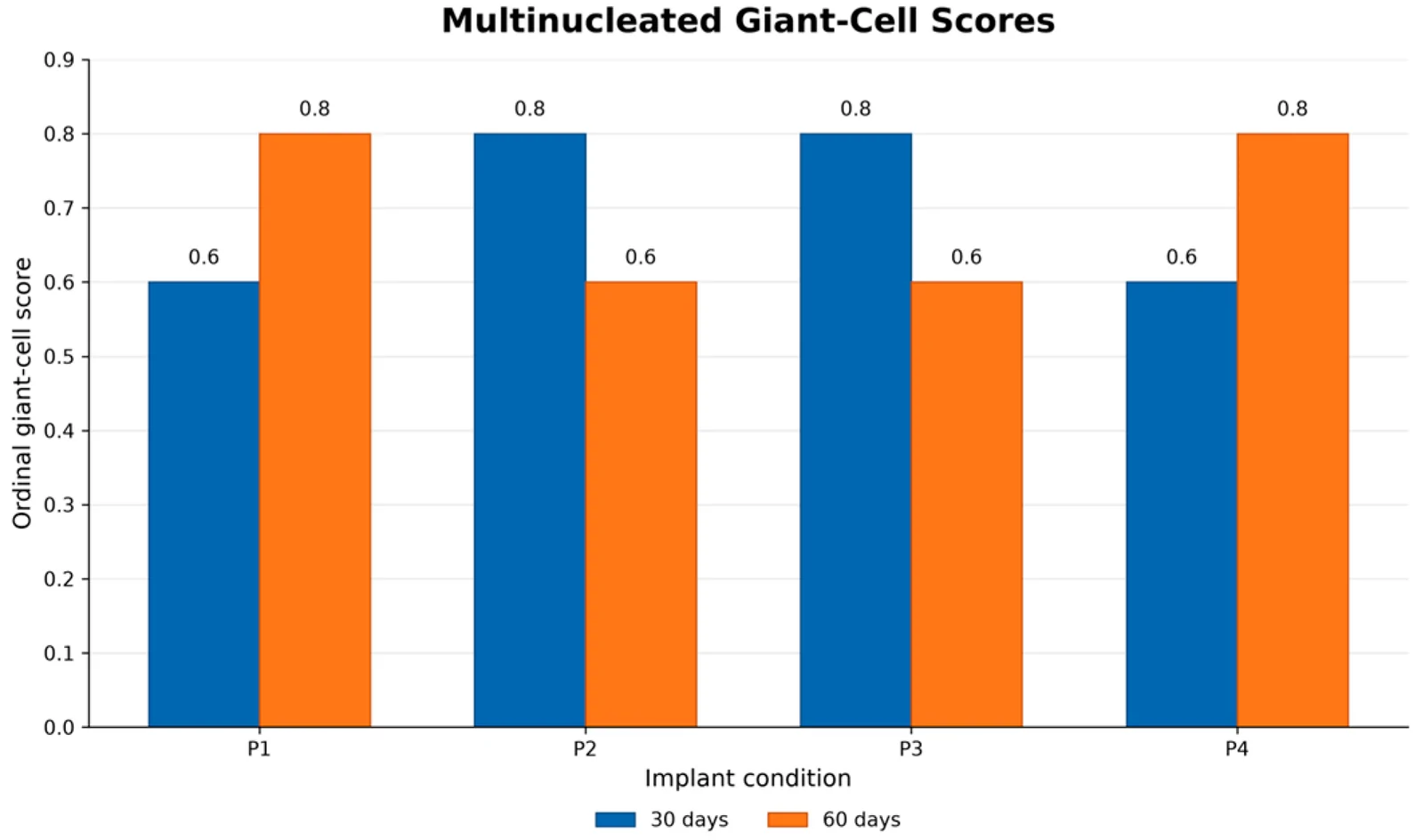
Multinucleated giant-cell scores according to implant condition and healing period. Blue bars/points represent the 30-day healing period, and orange bars/points represent the 60-day healing period.

Multinucleated giant cells were evaluated using an ordinal histological scoring system. Their occurrence across implant conditions and healing periods is presented in Figure 18. P1 and P4 showed scores of 0.6 at 30 days and 0.8 at 60 days, whereas P2 and P3 showed scores of 0.8 at 30 days and 0.6 at 60 days.

The observed multinucleated giant-cell scores were similar across several condition–period cells. With the small sample and fixed anatomical allocation, these findings do not establish equivalence or a coating-specific macrophage response.

#### 3.3.15. New Bone Formation: Maturity of Newly Formed Bone

**Figure 19:**
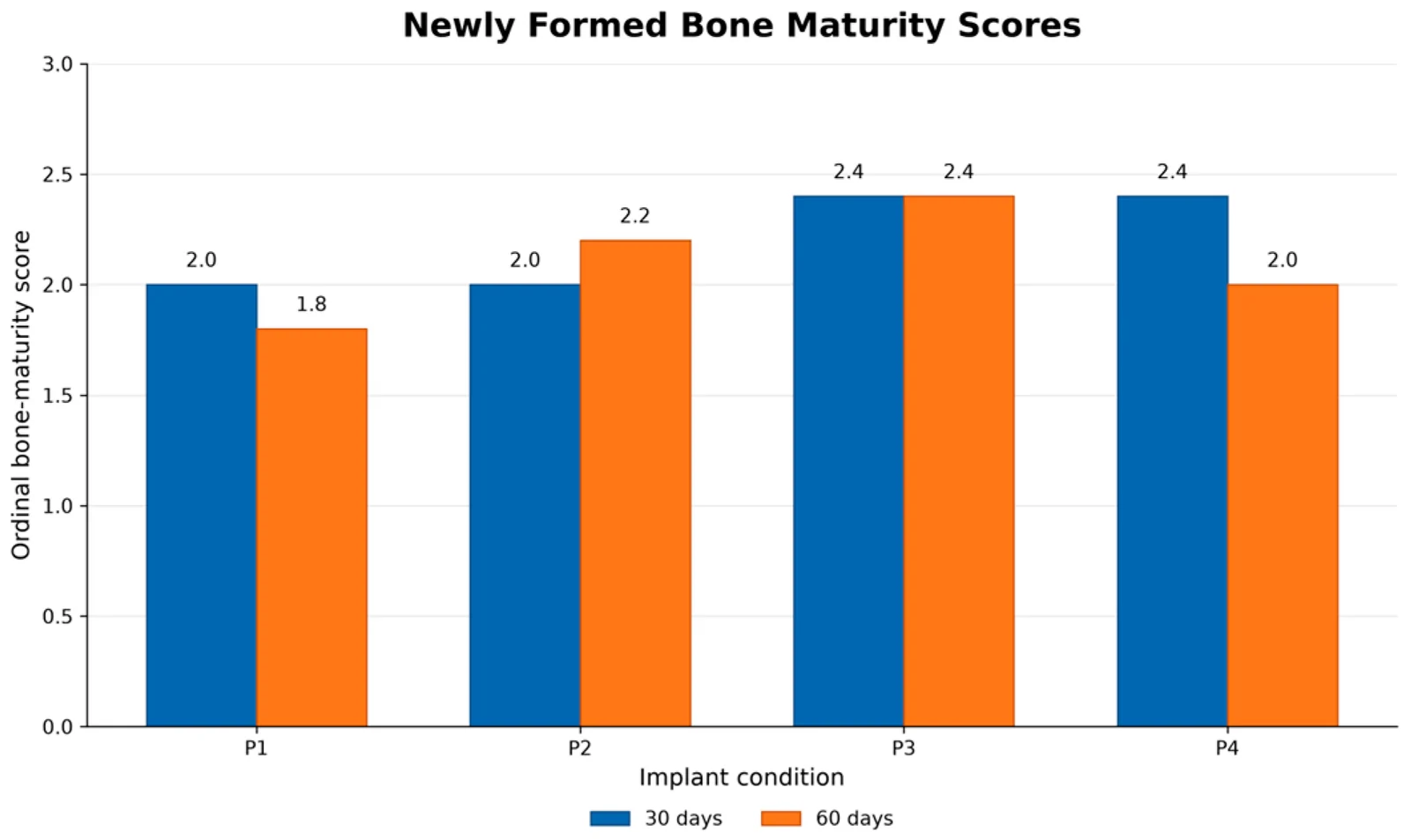
Newly formed bone maturity scores according to implant condition and healing period. Blue bars/points represent the 30-day healing period, and orange bars/points represent the 60-day healing period.

The maturity of newly formed bone was assessed using an ordinal score. Condition- and period-specific patterns are presented descriptively in Figure 19.

The ordinal scores provide a semi-quantitative description of the histological appearance of newly formed bone. They do not directly quantify the transition from woven to lamellar bone, osteoblast differentiation, mineral deposition, or extracellular matrix organization.

#### 3.3.16. Type I and Type III Collagen Proliferation Under Picrosirius Red Polarized Light

**Figure 20:**
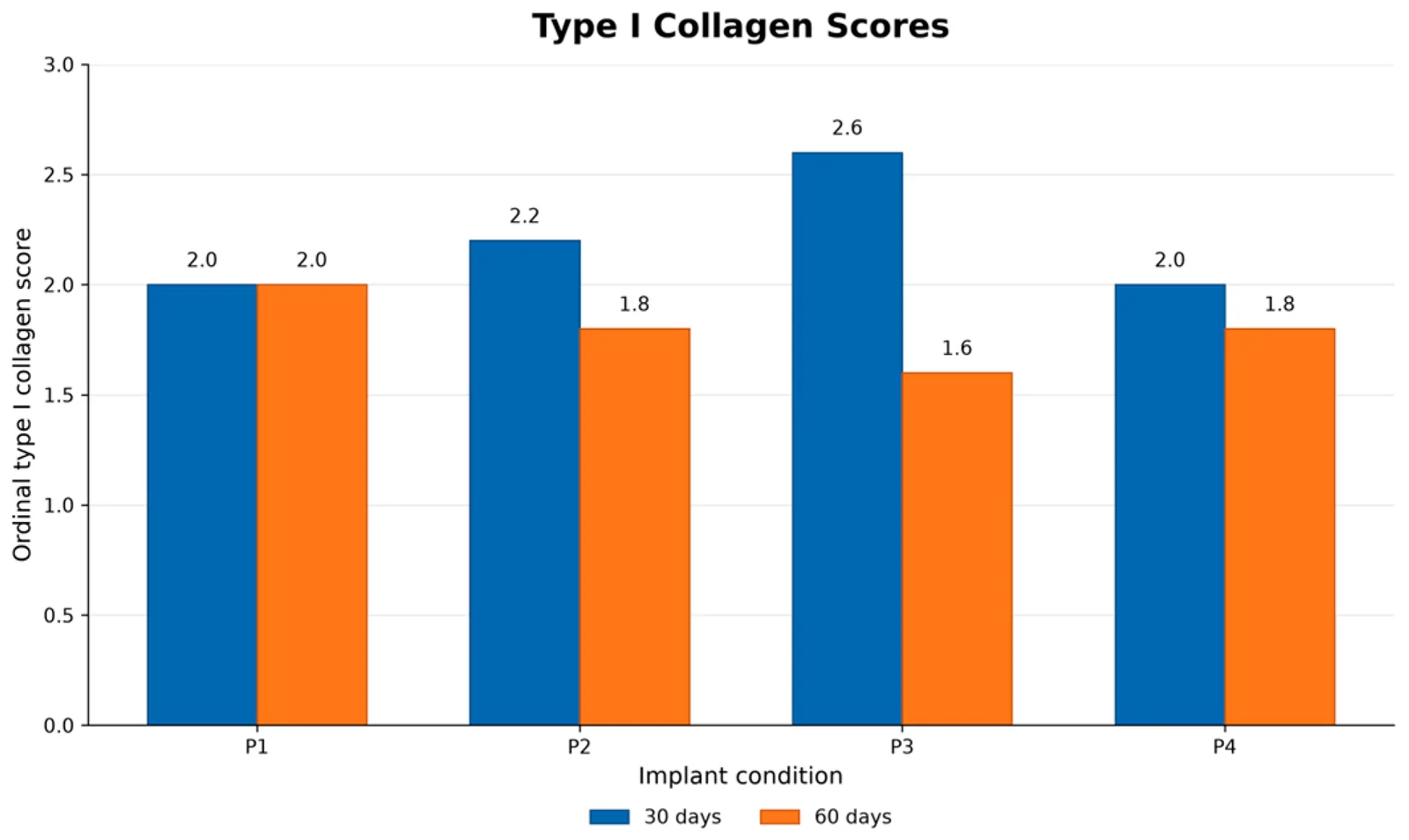
Type I collagen scores according to implant condition and healing period. Blue bars/points represent the 30-day healing period, and orange bars/points represent the 60-day healing period.

**Figure 21:**
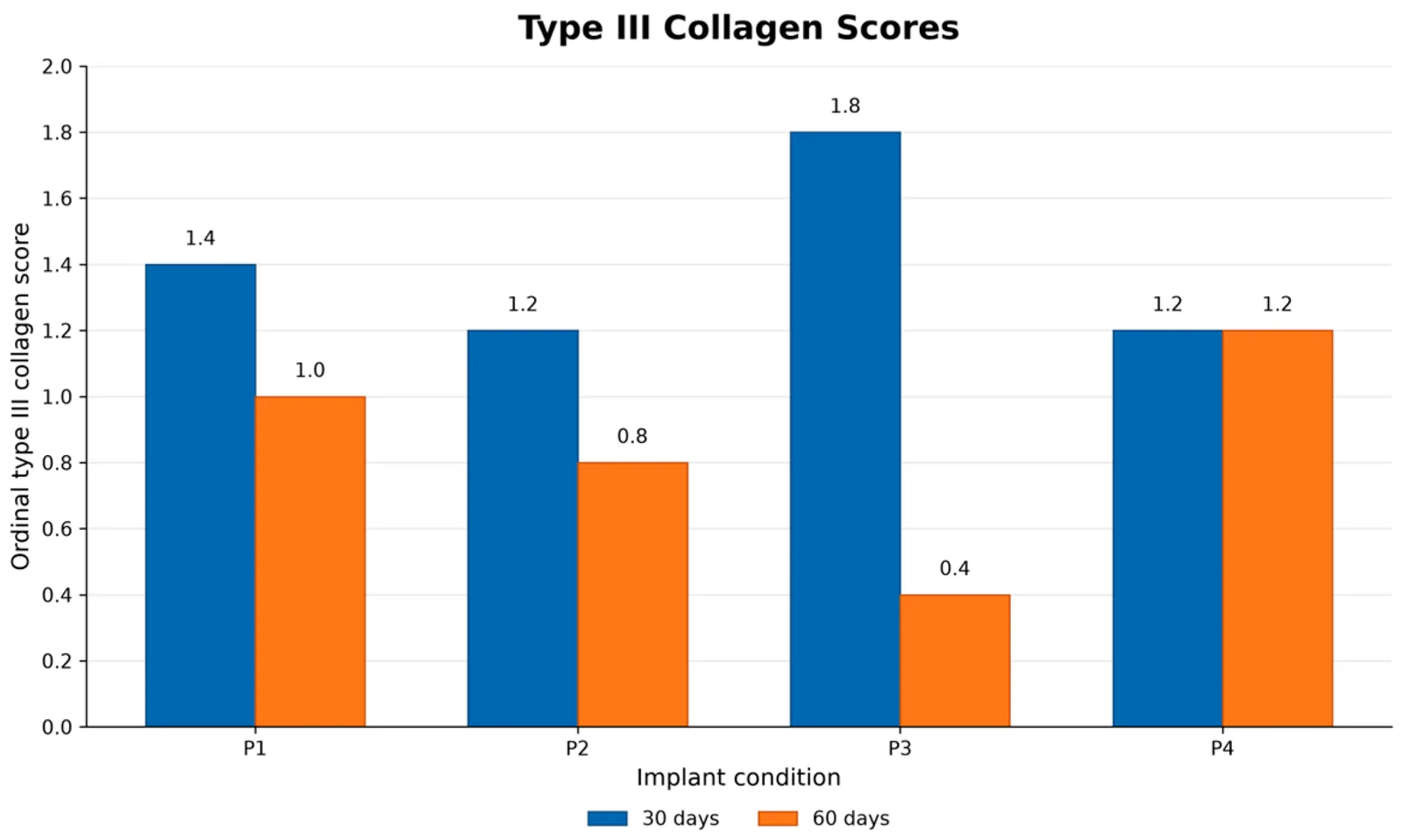
Type III collagen scores according to implant condition and healing period. Blue bars/points represent the 30-day healing period, and orange bars/points represent the 60-day healing period.

**Figure 22:**
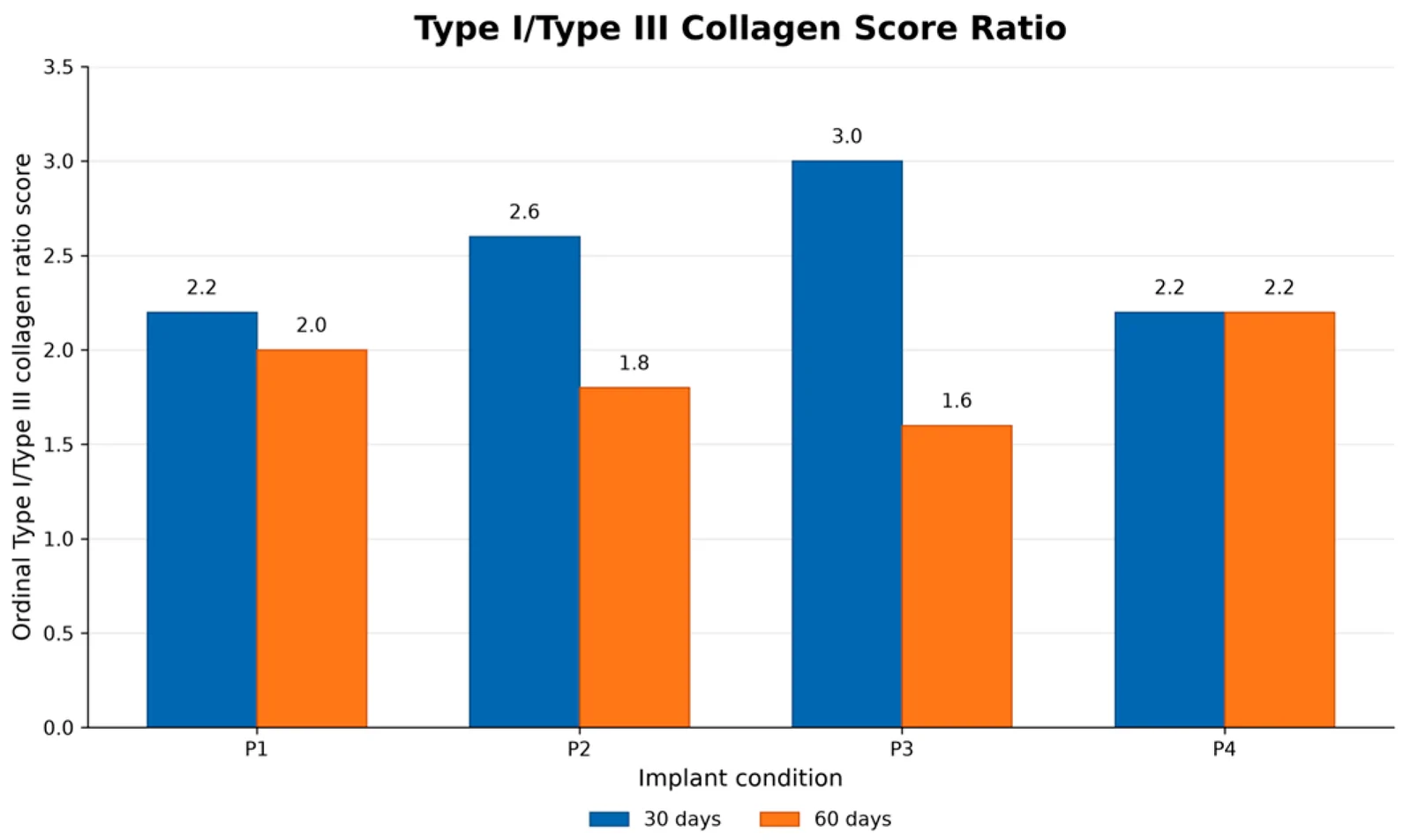
Type I/type III collagen ratio scores according to implant condition and healing period. Blue bars/points represent the 30-day healing period, and orange bars/points represent the 60-day healing period.

Picrosirius Red staining under polarized light was used to evaluate collagen fiber birefringence and organization. Orange-red- and green-predominant birefringence patterns were assessed using semi-quantitative ordinal scores and are presented according to implant condition and healing period in Figure 20 and Figure 21. These color patterns represent optical characteristics of collagen fiber organization and should not be interpreted as the molecular identification of collagen types I and III. The relative orange-red/green birefringence predominance score is presented in Figure 22. This score represents an optical semi-quantitative index and not a biochemical collagen I/III ratio.

The green-predominant birefringence scores are presented descriptively and do not provide direct evidence of collagen type III concentration or synthesis.

The type III collagen scores represent semi-quantitative histological observations and do not directly measure collagen concentration, molecular expression, or tissue repair kinetics. In this study, the type I/type III collagen ratio scores are presented in Figure 22. The graph presents the reported semi-quantitative ordinal scores obtained from Picrosirius Red staining under polarized light at 30 and 60 days.

The observed differences do not establish a generalized maturation trajectory or a coating-specific change in collagen composition. The scores should not be interpreted as direct biochemical ratios or quantitative measurements of collagen concentration.

## 4. Discussion

This exploratory in vivo study evaluated biomechanical, histological, and surface integrity responses associated with four titanium implant conditions over 30 and 60 days in a rabbit femoral condyle model.

Polarized-light examination of the Picrosirius Red-stained sections revealed differences in the optical appearance and organization of the peri-implant collagen matrix. These findings provide information about the structural arrangement and relative maturation of the fibers under experimental conditions. However, the observed color-dependent birefringence should not be interpreted as definitive molecular identification of collagen types, since collagen composition was not confirmed by immunohistochemistry, biochemical analysis, or molecular methods [17].

The principal findings were: (i) reverse-torque values increased over time in all conditions, with the highest mean values at 60 days observed for uncoated titanium (P2) and Graphene-DLC (P3), consistent with the use of reverse torque as a biomechanical indicator of implant anchorage and osseointegration in animal models [18]; (ii) peri-implant bone formation was observed across all conditions, although the histological outcomes showed heterogeneous temporal patterns; and (iii) surface integrity scores were lower in some niobium-containing conditions, indicating that coating stability deserves particular attention in the further development of these surface treatments. Previous studies have emphasized both the potential biomedical applications of diamond-like carbon coatings and the importance of coating composition, residual stress, adhesion, and environmental stability when interpreting their performance [19]. Similarly, niobium-based coatings have demonstrated promising physicochemical and biological properties, although their performance depends on the deposition method and resulting coating characteristics [20]. These findings should therefore be interpreted within the exploratory nature of the study and the predefined anatomical allocation of each implant condition.

The higher mean RTQ values observed in the 60-day cohort are compatible with greater fixation at the later endpoint, but the study did not follow the same animals longitudinally across time. Graphene-DLC (P3) and uncoated titanium (P2) each had a reported mean RTQ of 5.14 N.cm at 60 days. Exploratory mixed-effects modeling identified condition-by-period interactions for P2 and P3 relative to P1; however, because each surface treatment was fixed to one anatomical site, these coefficients represent combined implant-site conditions and cannot be attributed independently to coating chemistry. These observations support further investigation of the specific surface-processing conditions using randomized or counterbalanced anatomical allocation.

The niobium-containing conditions showed a different descriptive response pattern. Although the mean RTQ values increased between 30 and 60 days for both Nb-Gel (P1) and Nb-CVD (P4), surface integrity findings were more heterogeneous under the present protocol. Niobium- and niobium oxide-based treatments have been investigated because of their potential biocompatibility and effects on cell–material interactions [8,9,12]. However, these properties depend on oxide state, coating architecture, thickness, substrate preparation, and deposition technique. Nb-Gel and Nb-CVD should therefore be regarded as distinct surface treatments rather than interchangeable forms of a single niobium coating.

Because surface integrity was evaluated after reverse-torque testing and implant removal, the experimental sequence does not allow changes occurring during the in vivo period to be distinguished from those potentially induced or accentuated during mechanical testing. Reverse-torque analysis is an invasive and potentially destructive method; the applied torque may disrupt the bone–implant interface and alter the original state of the osseointegrated specimen [21,22]. The present findings therefore identify coating stability as a relevant target for further investigation but do not establish spontaneous in vivo delamination. The lower integrity scores observed in some niobium-containing specimens raise an important technical question regarding coating adhesion and mechanical stability. Coating performance depends not only on the chemical composition of the deposited layer but also on the interfacial adhesion, residual stress, substrate compatibility, thickness, and stability under physiological conditions [23].

Histological findings demonstrated peri-implant tissue responses across all conditions, with heterogeneous patterns between 30 and 60 days. Time-dependent differences in bone formation, remodeling, bone-to-implant contact, and tissue organization are expected during peri-implant healing and may vary according to the surface topography, local bone conditions, and biological stage of repair [24]. In the present study, osteoclastic-proliferation scores, BIC scores, peri-implant interface thickness, bone organization, osteoid formation, tissue maturation, vascular response, inflammatory infiltrate, and newly formed bone varied among conditions and healing periods. Taken together, these findings demonstrate ongoing peri-implant remodeling but do not reveal a uniform histological trajectory favoring a single surface treatment.

Surface characterization also represents an important methodological consideration. Raman spectroscopy and X-ray photoelectron spectroscopy (XPS) were performed on reference specimens produced under the same respective coating conditions as those of the experimental implants rather than on the implanted devices themselves. These techniques provide valuable information regarding vibrational features, elemental composition, and surface chemical states; however, when performed on reference specimens, they cannot document alterations that may have occurred on the individual implants during implantation, mechanical loading, tissue contact, or retrieval. Surface characterization of titanium-based implants is most informative when chemical data are integrated with morphological, topographical, dimensional, and mechanical assessments because implant performance is influenced by the interaction of these properties rather than by surface chemistry alone [25,26].

Comprehensive measurements of surface roughness, morphology, coating thickness, adhesion strength, and post-implantation particle release were not available in the present study. Coating adhesion should be assessed using a standardized mechanical method. Scratch testing, for example, can provide comparative information regarding coating adhesion, although the measured critical load should not automatically be interpreted as an absolute interfacial adhesion strength [27]. Accordingly, the present Raman and XPS findings should be interpreted as the characterization of representative coating conditions rather than as direct evidence of the post-implantation state of every retrieved implant. Future studies should combine chemical characterization with morphological, topographical; thickness, particle release, and standardized adhesion measurements performed on well-defined specimens from the same experimental coating batches.

Another methodological consideration is the sequence of specimen processing. Bone–implant specimens were fixed in formalin for two weeks before reverse-torque testing, and the same specimens were subsequently processed for histological evaluation. Chemical fixation can modify the mechanical behavior of bone by altering the collagenous matrix and tissue hydration; therefore, biomechanical measurements obtained after fixation may not be directly equivalent to measurements obtained from fresh specimens [28]. In addition, reverse-torque testing and implant removal are inherently invasive procedures that may mechanically alter the bone–implant interface and coating surface. Consequently, BIC, interface morphology, and surface integrity observations should be interpreted within this experimental sequence rather than as measurements of an undisturbed in vivo interface. Future studies should consider separate, prospectively allocated specimens for biomechanical and histological analyses and should standardize the preservation protocol across all experimental groups.

A further limitation concerns the systematic anatomical allocation of implant conditions. Because each surface treatment was consistently assigned to the same femoral condyle site, potential effects related to laterality and medial-versus-lateral position cannot be fully separated from those associated with surface treatment. The between-conditioning findings should therefore be interpreted as exploratory associations between combined implant-site conditions rather than as unbiased estimates of coating-specific effects. In animal experiments, randomization, allocation concealment, and blind outcome assessment are important design features because their absence may increase the risk of systematic bias [29]. Future confirmatory studies should use randomized or counterbalanced allocation while retaining the within-animal design and should report the allocation procedure in accordance with current animal research reporting recommendations [30].

Despite these limitations, this study provides an integrated biomechanical, histological, and surface integrity assessment of three distinct surface modification strategies compared with uncoated titanium. The findings generate two hypotheses for future investigation: first, that the Graphene-DLC treatment may be associated with favorable biomechanical fixation during early osseointegration; and second, that the performance of niobium-containing coatings may depend on not only their biological properties but also on coating architecture, interfacial adhesion, thickness, residual stress, and mechanical stability. The design and architecture of an implant coating can influence its biological response and its resistance to mechanical and physiological challenges, so chemical composition alone may not adequately predict in vivo performance [31]. Randomized or counterbalanced studies with larger samples, comprehensive surface characterization, and independent specimens for biomechanical and histological analyses are warranted to test these hypotheses.

## 5. Conclusions

This exploratory in vivo study demonstrated peri-implant bone formation across all evaluated titanium implant conditions, with distinct temporal patterns in biomechanical, histological, and surface integrity outcomes between 30 and 60 days. Mean reverse-torque values were higher in the 60-day cohort than those in the 30-day cohort in all conditions, with the highest reported mean values at 60 days observed for uncoated titanium (P2) and Graphene-DLC (P3). Histological evaluations demonstrated heterogeneous patterns of bone-to-implant contact, osteoclastic activity, bone organization and maturation, peri-implant interface thickness, inflammatory response, and collagen fiber birefringence.

Surface integrity assessment revealed lower reported integrity scores in some niobium-containing implant conditions. These findings raise the possibility that coating adhesion and mechanical stability may be important factors in the performance of the specific niobium-based treatments evaluated. However, because surface integrity was assessed after reverse-torque testing of the same specimens, the present sequence does not allow changes occurring in vivo to be distinguished from those potentially induced or accentuated during mechanical testing and implant removal.

The experimental design systematically assigned each implant surface to a predefined femoral condyle site rather than randomizing allocation among anatomical locations. Potential anatomical-site effects therefore cannot be completely separated from surface treatment effects. Between-condition differences should be interpreted as exploratory associations rather than definitive evidence of coating-specific superiority. Overall, the combined findings support further investigation using randomized or counterbalanced anatomical allocation, larger samples, clustered animal-level analysis, comprehensive coating characterization, and independent specimens for biomechanical and histological analyses.

## 6. Patents

System, method, and packaging for electro-energized implants with promotion of tissue repair and osseointegration and corresponding packaging. BR 10 2026 019764 5 [32].

## Figures and Tables

**Table 1:** Reverse-torque values (N.cm) according to implant condition and healing period.

Condition	Day	*n*	Mean ± SD	Median	Range
P1	30	5	3.46 ± 0.58	3.20	3.2–4.5
P1	60	5	4.08 ± 1.82	3.20	2.0–6.0
P2	30	5	2.48 ± 0.66	2.00	2.0–3.2
P2	60	5	5.14 ± 1.26	6.00	3.2–6.0
P3	30	5	2.98 ± 1.04	3.20	2.0–4.5
P3	60	5	5.14 ± 1.26	6.00	3.2–6.0
P4	30	5	3.22 ± 0.88	3.20	2.0–4.5
P4	60	5	4.08 ± 1.82	3.20	2.0–6.0

## Data Availability

The original contributions presented in the study are included in the article, further inquiries can be directed to the corresponding author.
